# Deep eutectic solvents based on matrine and menthol: A novel and safe transdermal penetration enhancer

**DOI:** 10.1016/j.ijpx.2026.100535

**Published:** 2026-04-03

**Authors:** Hongdou He, Xinyu Huang, Yi Hong, Zhenpeng Qiu, Fei Xu, Shan Lu, Yujie Guo

**Affiliations:** a Research Center for Pharmaceutical Preparations, School of Pharmacy, Hubei University of Chinese Medicine, Wuhan 430065, China; b Hubei Shizhen Laboratory, Wuhan 430061, China; c Institute of Feed Research, Chinese Academy of Agricultural Sciences, Beijing 100081, China; d Hubei Key Laboratory of Resources and Chemistry of Chinese Medicine, School of Pharmacy, Hubei University of Chinese Medicine, Wuhan 430065, China; e Center of Traditional Chinese Medicine Modernization for Liver Diseases, Hubei University of Chinese Medicine, Wuhan 430065, China

**Keywords:** Deep eutectic solvents, Transdermal delivery, Matrine, Menthol, Molecular mechanism, Theoretical calculations

## Abstract

Transdermal drug delivery has attracted extensive attention due to its ability to bypass the first-pass metabolism, maintain plasma drug concentrations, and improve patient compliance. However, the transdermal delivery of hydrophilic drugs is still quite challenging. This study aims to develop a novel deep eutectic solvent (DES) composed of matrine (MT) from *sophora flavescens Aiton* and menthol (Men) from *Mentha canadensis* to enhance the transdermal delivery of hydrophilic drugs. Geniposide (GS) was selected as the model drug. Combining multiple experimental techniques and theoretical calculations, it was demonstrated that the formation of MT-Men DES was primarily driven by hydrogen bonding, with van der Waals forces playing a supplementary role. *In vitro* penetration tests showed that the MT-Men (1:1) DES significantly enhanced the cumulative permeation percentage of GS, achieving a 5.77-fold increase compared to the GS aqueous solution. *In vivo* pharmacokinetic results indicated that MT-Men (1:1) DES significantly increased the transdermal bioavailability of GS by 2.32-fold and sustained permeation over 24 h. In a rheumatoid arthritis (RA) model, GS@MT-Men (1:1) DES effectively exerted anti-inflammatory effect. Skin irritation and cytotoxicity tests indicated that both MT-Men (1:1) DES and GS@MT-Men (1:1) DES exhibited excellent biocompatibility and safety for the skin. In conclusion, this study innovatively develops a novel and safe transdermal penetration enhancer that might help address the challenges of transdermal delivery of hydrophilic drugs.

## Highlights

• Constructing various DES and analyzing their related properties and molecular mechanisms.
• Designing a safe and efficient transdermal penetration enhancer for GS.
• MT-Men (1:1) DES increased the transdermal bioavailability of GS by 2.32-fold.
• GS@MT-Men (1:1) DES can effectively replace oral medication in the treatment of RA.

## Graphical abstract

**Figure:**
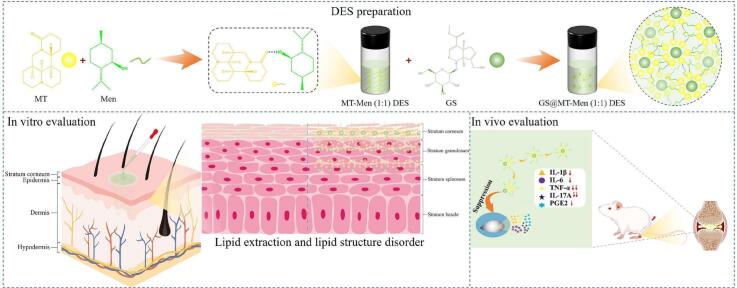

## 1 Introduction

Transdermal drug delivery is considered the most promising method compared to oral and injectable administration. It not only significantly improves patient compliance and the convenience of self-administration but also bypasses the first-pass effect in the liver, enhances drug bioavailability, alleviates gastrointestinal reactions, and enables sustained delivery (Qindeel et al., 2020). However; the outermost protective layer of the skin; composed of a ‘brick and mortar’ structure like the stratum corneum formed by tightly arranged intercellular lipids and hydrated keratin; limits the permeation of most drugs (Lefèvre-Utile et al., 2021). Drugs are primarily classified into hydrophilic and hydrophobic categories based on their solubility. Compared to hydrophobic drugs; hydrophilic drugs face greater challenges in transdermal delivery due to their poor skin permeability. In recent years; The techniques used to address the transdermal permeability of hydrophilic drugs mainly include: design of oil-miscible ionic liquid (Md Moshikur et al., 2022); Preparation of microemulsions (Yadav et al., 2023); and construction of solid-in-oil nano dispersion systems (Zhang et al., 2023). However; many issues related to drug-loading performance; biocompatibility; and biosafety still exist. For example; the use of high concentrations of surfactants or co-surfactants may cause irritation or toxicity to the skin (Schmidts et al., 2011). In addition; the complex preparation processes and production costs make these systems less ideal as delivery carrier (Gu et al., 2018; Schlich et al., 2022).

In contrast, deep eutectic solvents (DES) avoid the biocompatibility and safety issues associated with certain carriers. DES are eutectic mixtures formed by mixing hydrogen bond acceptors (HBAs) and hydrogen bond donors (HBDs) in a specific molar ratio, resulting in a melting point lower than room temperature (Hansen et al., 2021). Both HBAs and HBDs involved in the composition of DES are natural small molecule compounds (Liu et al., 2018). DES exhibit numerous advantages; including ease of preparation; high biocompatibility; low toxicity; and high biodegradability (Hansen et al., 2021). As a novel carrier; the application value of DES in transdermal drug delivery is becoming increasingly prominent. For example; Li et al.; used an oxymatrine-fatty acid DES system as a carrier to effectively enhance the transdermal permeation of the poorly soluble drug quercetin (Li et al., 2023). In addition; He et al.; constructed a THDES system using mirtazapine and medium-chain fatty acids to enhance the transdermal delivery of the poorly soluble drug mirtazapine(He et al., 2025). Moreover; Yang et al.; used a matrine-lauric acid DES system to enhance the solubility and stability of curcumin; and this DES system can spontaneously form a DES gel in water to improve the transdermal permeability of curcumin (Yang et al., 2024). Xie et al.; achieved transdermal delivery of water-insoluble Amphotericin B through a choline-geranate DES system (Xie et al., 2025). However, these studies focused on enhancing the transdermal permeation of hydrophobic drugs. In fact, so far, there is a gap in research regarding the construction of DES for the transdermal delivery of hydrophilic drugs.

Matrine (MT) is a quinolizidine alkaloid isolated from the root of *Sophora flavescens Aiton*, which has been demonstrated to effectively treat skin inflammation, pruritus, and allergic diseases (Gong et al., 2016; Wang et al., 2025). From a molecular structure perspective, the carbonyl group in its ring exhibits a certain ability to accept protons, indicating that it acts as a proton receptor for intermolecular hydrogen bonds. Menthol (Men), the main component in *Mentha piperita*, is a monoterpene organic compound with potential analgesic activity and can be used to alleviate pain (Mahboubi, 2017). Additionally; due to its minimal toxicity and irritation to the skin; Men has been widely used as a skin penetration enhancer to improve the transdermal permeation of drugs such as quercetin (Huang et al., 2019); Osthole (Yang et al., 2016); and 5-fluorouracil (Wang et al., 2017). Related studies indicate that the permeation-promoting effect of Men is superior to that of other terpene compounds; such as terpineol; menthone; pulegone; and carvone (Jain et al., 2002). Given the hydroxyl group in its molecular structure, it suggests the potential to act as a proton donor and participate in the formation of intermolecular hydrogen bonds.

**Scheme 1:**
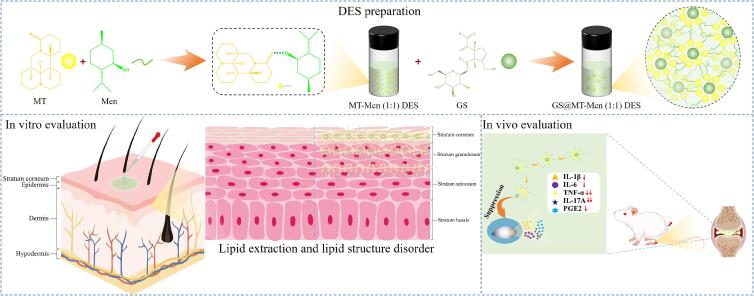
Schematic illustration of the formation of GS@MT-Men (1:1) DES, the mechanism by which MT-Men (1:1) DES promotes the transdermal delivery of GS, and the inhibitory effect of GS on inflammatory cytokine secretion by immune cells.

Therefore, in this work, MT from *Sophora flavescens* and Men from *Mentha piperita* were first reported to prepare DES for enhancing the transdermal delivery of GS. Different molar ratios of DES were first prepared, and then polarizing microscope (POM) and differential scanning calorimetry (DSC) measurements were used to confirm the amorphous nature of the DES. Thermogravimetric (TG) analysis was performed to evaluate the thermal stability of the DES. Fourier transform infrared spectroscopy (FTIR) and nuclear magnetic resonance (^1^H NMR) were used to demonstrate that the formation mechanism of DES primarily involves the generation of intermolecular hydrogen bonds. Molecular dynamics (MD) simulations and density functional theory (DFT) calculations further explained the formation of hydrogen bonds. The viscosity of the DES was measured using a rheometer, and the optimal molar ratio was selected through solubility experiments for *in vitro* release and permeation tests. The permeation mechanism of DES was then studied using ATR-FTIR. Finally, the MT-Men (1:1) DES with the best permeation enhancement effect underwent *in vivo* pharmacokinetic studies and an evaluation of its therapeutic effect on rheumatoid arthritis (RA). In addition, to further prove the safety of MT-Men (1:1) DES, cytotoxicity and skin irritation tests were also performed. In conclusion, our research findings provide an important reference for developing the novel and safe transdermal penetration enhancers. (Scheme 1).

## 2 Materials and methods

### 2.1 Materials

Matrine (MT, ≥98%) was purchased from Shanghai Macklin Biochemical Technology Co., Ltd. (Shanghai, China). Menthol (Men, ≥98%) and geniposide (GS, ≥98%) were purchased from Shanghai Yuanye Bio-Technology Co., Ltd. Chromatographic-grade methanol and acetonitrile were purchased from Sigma-Aldrich (Shanghai, China). Cy3-geniposide was purchased from Ruixi Biotechnology Co., Ltd. HaCat cells, minimum essential medium (MEM (with NEAA)), and fetal bovine serum (FBS) were purchased from Wuhan Pricella Biotechnology Co., Ltd. (Wuhan, China). Porcine skin was purchased from Shanghai Xinlaituo Biotechnology Co., Ltd. (Shanghai, China). Biomimetic membranes of Strat-M™ were purchased from Millipore Corp. (MA, USA). Complete Freund's adjuvant (CFA) was purchased from Chondrex (WA, USA). All the other chemicals were of analytical grade and used without further purification.

### 2.2 Preparation of DES

A series of DES samples were prepared by mixing MT from *Sophora flavescens* with Men from *Mentha piperita* at different molar ratios. The specific preparation method follows previous reports (He et al., 2025). The MT and Men were separately weighed according to their molar ratio and placed in sample bottles at room temperature (25 °C). A small amount of anhydrous ethanol was added to the sample bottles and stirred for 30 min until the liquid became clear. The anhydrous ethanol was then removed by rotary evaporation at 45 °C until the liquid volume stabilized and no longer decreased. Finally, the product was dried in a vacuum drying oven at 30 °C and − 1 bar (DZF-6020, Shanghai Yiheng Scientific Instrument Co., Ltd.) for 48 h until a constant weight was achieved. The specific experimental parameter values are shown in Table 1.

**Table 1:** Summary of instrument parameters used in the preparation of MT-Men DES with different molar ratios.

	Molar ratios	stirring time (min)	stirring speed (rpm)	Evaporation time (min)
MT-Men DES	1:1	30	200	20
	1:2	30	200	20
	1:3	30	200	20
	1:4	30	200	20
	1:5	30	200	20

### 2.3 Characterization of DES

#### 2.3.1 Tyndall effect observation

A certain volume of DES samples, water, and ethanol was placed in the different glass sample bottles, and then vertically illuminate them using a laser pointer (303r, Beijing Huisite Technology Co., Ltd., China).

#### 2.3.2 Polarizing microscope observation

The prepared DES samples and their pure components were observed under dark field conditions using a polarized light microscope (ZML310, Shanghai Zimeng Technology Co., Ltd., China). The procedure involves placing a small amount of the sample to be observed on a glass slide, followed by adding a small amount of white mineral oil to enhance the observation effect. To ensure uniform distribution of the sample, a coverslip is placed over it, and the objective lens is adjusted to 20× for observation.

#### 2.3.3 Differential scanning calorimetry

Standard substances (indium and zinc) were used to calibrate the DSC3 (METTLER TOLEDO, Switzerland). Then, approximately 3 to 10 mg of MT, Men, and the prepared DES samples were weighed and placed in crucibles. These crucibles were sealed using a crucible sealing press. Finally, they were placed in the DSC furnace and subjected to a programmed heating rate of 10 °C/min. The testing temperature ranges for the pure components and DES samples were 25 to 240 °C and − 80 to 150 °C, respectively, with additional nitrogen protection during testing. The results were analyzed and processed using STARe software.

#### 2.3.4 Thermogravimetric analysis

Approximately 5–8 mg of MT, Men, and the prepared DES samples were weighed and placed in a crucible. The crucible was then placed in the furnace of the TGA3^+^ (METTLER TOLEDO, Switzerland) thermogravimetric analyzer. A programmed temperature increase from 40 to 500 °C was conducted at a rate of 10 °C/min, with nitrogen protection during the test. The results were analyzed and processed using STARe software.

#### 2.3.5 Fourier transform infrared spectroscopy

The samples were analyzed using FTIR (Nicolet6700 Intelligent Fourier Transform Infrared Spectrometer, Thermo Scientific) within the range of 4000–400 cm^−1^. For MT and Men, the potassium bromide (KBr) pellet method was employed. In brief, approximately 12 mg of solid samples were weighed, finely ground in an agate mortar, and then thoroughly mixed with dried KBr in a 1:100 ratio before being compressed into tablets. The tablets were then placed in the sample chamber of the spectrometer for 32 scans. For the DES samples, a liquid film method was employed. The DES samples were directly applied onto a KBr crystal plate for 32 scan tests.

#### 2.3.6 ^1^H NMR analysis

15 mg of DES samples or pure components were dissolved in 0.5 mL of DMSO‑*d*_6_ and then quickly transferred to a 5 mm NMR tube. ^1^H NMR spectra were recorded using an Avance III HD 400 MHz nuclear magnetic resonance spectrometer (Bruker, Germany). The final spectra data were analyzed using MestReNova software.

#### 2.3.7 Rheological measurement of DES

The rheological properties of the DES samples were evaluated using a Visco Tester iQ Air rheometer (Thermo Fisher Scientific, Germany). Before testing, the instrument was calibrated by adjusting the gap between the parallel plates to 1 mm. Then, 1 mL of the DES sample was placed on the bottom plate, and a programmed temperature increase from 25 to 45 °C was performed, with measurements taken every 5 °C and a 100-s equilibration period at each corresponding temperature. In controlled rate (CR) mode, the shear rate was changed from 0.1 s^−1^ to 150 s^−1^, recording 60 data points. The data were analyzed using Rheo Win Data Manager software.

### 2.4 MD simulations and DFT calculations

Molecular dynamics simulations were performed using Material Studio 2019 software, with the force field type set to COMPASS II. All molecular structures were obtained from the PubChem database. A cubic box with periodic boundaries for MT-Men (1:1) DES was constructed using the amorphous cell module at 298 K. The smart algorithm was then applied for 5000 iterations to ensure the lowest energy configuration for each system. Subsequently, 5.0 ns of NPT (constant particle number, temperature, and pressure) and NVT (constant particle number, volume, and temperature) molecular dynamics simulations were performed. The time step was set to 1 fs, and the Nose thermostat and Berendsen barostat were used to maintain a constant temperature of 298 K and a pressure of 1 atm, respectively. Calculations of van der Waals forces and electrostatic forces were performed using atom-based summation and particle-mesh Ewald summation, respectively. A frame was output every 10 ps, resulting in a total of 501 frames (including the initial one) of trajectory files for the analysis of the radial distribution function (RDF).

Quantum chemical calculations based on density functional theory (DFT) were performed using Gaussian 16 W and Gauss View 6.0 software. The most likely binding modes between MT and Men molecules were designed, and the B3LYP/6-31G (d, p) basis set was used for geometry optimization and frequency calculations, with the addition of the em = gd3bj dispersion correction. All species were fully optimized without any imaginary frequencies. Additionally, basis set superposition error (BSSE) correction was performed using the Gaussian program. The calculation formula for the corrected intermolecular interaction energy is as follows:

(1) $$\Delta E_{\mathrm{alkaloid}-\mathrm{Men}}=E_{\mathrm{alkaloid}-\mathrm{Men}}-\left(E_{\mathrm{alkaloid}}+E_{\mathrm{Men}}\right)+\mathrm{EBSSE}$$

Where *E*_*alkaloid−Men*_ represents the total energy of the complex formed between the alkaloid and men, while *E*_*alkaloid*_ and *E*_*Men*_ are the energies of the isolated alkaloid and Men, respecti*v*ely.

In addition, electrostatic potential (ESP) and the independent gradient model based on Hirshfeld partition (IGMH) were calculated using the Multiwfn 3.8 program (Lu, 2024; Lu and Chen, 2012; Lu and Chen, 2022).

### 2.5 Establishment of the content method for GS using HPLC

LC-20 A HPLC instrument (Shimadzu, Japan) was used to determine the content of GS, with the Diamonsil C18 (4.6 × 250 mm, 5 μm) column selected. The mobile phase consists of 0.1% phosphoric acid aqueous solution and acetonitrile, with a ratio of 82:18 (*v*/v). The UV detection wavelength and flow rate were set to 239 nm and 1.0 mL/min, respectively. The column temperature and injection volume were set to 35 °C and 10 μL, respectively, and the single-needle sample collection time was 10 min. The calibration curve equation for GS is y = 13,620× - 1940.9 (R^2^ = 0.9998).

### 2.6 Determination of the solubility of GS in DES

The saturated solubility of GS was determined using the shaking flask method. An excess amount of GS was added to 0.5 mL of DES, and shaken continuously at a constant temperature of 32 ± 0.5 °C in a water bath shaker for 48 h. The supersaturated solution was then filtered using a 0.45 μm nylon filter, and the filtrate was diluted with methanol to an appropriate concentration and analyzed using HPLC equipped with a UV–Vis detector (Shimadzu, Japan).

### 2.7 *Ex vivo* permeability study of GS

#### 2.7.1 Preparation of GS@MT-Men (1:1) DES samples

Approximately 50 mg of GS was weighed and placed in 5 mL of MT-Men (1:1) DES, then sonicated for 30 min to obtain the target GS@MT-Men (1:1) DES samples.

#### 2.7.2 *In vitro* release test

*In vitro* release test was performed using a dry heating transdermal diffusion instrument (DHC-6 T, LOGAN, USA), which was equipped with diffusion cells having a receptor volume of 12.0 mL and a diffusion area of 1.77 cm^2^. A magnetic stirrer was first placed into the receiver cell, which was then filled with a certain volume of PBS buffer solution with a pH of 7.4 (since GS in the test sample is a hydrophilic component, the PBS buffer solution satisfies the sink conditions). Strat-M™ membranes were then fixed between the receiver and diffusion cells, with the shiny surface facing the donor compartment. 200 mg of the GS@MT-Men (1:1) DES sample was evenly spread on the smooth side of the membrane. The entire experiment was conducted at 32 ± 1 °C and 600 rpm. At specified time points (0.5, 1, 2, 4, 6, 8, 10, 12, and 24 h), 4 mL of solution was taken from the receiver cell, and an equal volume of isothermal fresh PBS buffer solution was added simultaneously. The cumulative release amount per unit area was determined using high-performance liquid chromatography (HPLC). The specific calculation formula is as follows:

(2) $$\begin{array}{c} Q_{n}=\frac{C_{n}V_{r}+\sum_{i=0}^{n-1}C_{i}V_{s}}{A} \end{array}$$

where *C*_*n*_ and *C*_*i*_ are the concentrations of GS at the *n*th and *(n − 1)*th sampling points, respectively, while *V*_*r*_ and *V*_*s*_ represent the total volume of the receiver cell and the sampling volume, respectively. *A* is the diffusion area.

#### 2.7.3 *Ex vivo* permeation experiments

We first purchased fresh porcine skin from a local supplier (Shanghai Xinlaituo Biotechnology Co., Ltd.), then cut it into 2.5 × 2.5 cm squares and soaked them in a beaker containing PBS buffer solution with a pH of 7.4 at a constant temperature of 32 °C for 30 min. The unused porcine skin was stored in a freezer at −40 °C. The experiment was conducted using the same instruments as in the *in vitro* release test. In brief, 12 mL of PBS buffer solution (pH 7.4) was added to the receiver cell, and fresh porcine skin was fixed between the receiver and diffusion cells, ensuring the stratum corneum faced upwards. Approximately 15 mg of GS@MT-Men (1:1) DES samples was evenly applied to the stratum corneum. At fixed time points (0.5, 1, 2, 4, 6, 8, 10, 12, and 24 h), 4 mL of receiving solution was taken from the receiver cell, and an equal volume of isothermal fresh PBS buffer (pH 7.4) was added to replenish it. The cumulative permeation amount per unit area was then calculated using the same method as the *in vitro* release test.

#### 2.7.4 Skin retention amount investigation

After the *in vitro* permeation test was completed, the porcine skin was removed, washed with PBS buffer solution (pH 7.4), and then cut into pieces. Methanol ultrasonic extraction was performed for 30 min. Finally, the extract was filtered through a 0.45 μm filter membrane, and the filtrate was analyzed using HPLC to determine the GS content in the skin layers.

### 2.8 Effect of DES on the *ex vivo* skin

#### 2.8.1 Determination of ATR-FTIR

After the *ex vivo* permeation test, the porcine skin was removed, carefully washed with PBS buffer solution (pH 7.4) to remove any remaining sample from the surface, and then placed in a desiccator to dry for 72 h (Given that the moisture in fresh skin tissue can interfere with signal collection, the samples were subjected to a drying process). Finally, the stratum corneum of the porcine skin was positioned facing down on the ATR crystal of the FTIR spectrometer (Nicolet iS50-Continuum, Thermo Fisher), and spectra were collected in the range of 4000–400 cm^−1^.

#### 2.8.2 Determination of fluorescence microscope

The same treatment method as the *ex vivo* permeation test was used. In brief, fresh porcine skin was fixed between the diffusion cell and the receiver cell, with the stratum corneum facing upward. The receiving medium was PBS buffer solution (pH 7.4), with a magnetic stirrer in place. Approximately 15 mg of cy3-GS@MT-Men (1:1) DES (containing 150 μg of GS) was evenly applied onto the skin's stratum corneum. After 24 h of permeation, the skin was removed and cut into 20 μm slices using a cryostat (CRYOSTAR NX50, Thermo). The slices were then examined under a fluorescence microscope (NIKON ECLIPSE E100, Japan) for fluorescence imaging.

### 2.9 *In vivo* pharmacokinetic studies of GS

#### 2.9.1 Grouping and administration

Sprague-Dawley (SD) rats (male, 6–8 weeks old, weighing 200 ± 20 g) were purchased from the Experimental Animal Center of Ocean University of China (Certification No. SCXK 2022 0006). The study was approved by the ethics committee (approval number: OUC-SMP-2024-10-03). Prior to the experiment, the rats were allowed 3–7 days for adaptive feeding. During this period, all procedures were strictly performed in accordance with the ‘Regulations on the Administration of Laboratory Animals’ and approved by the Animal Subject Review Committee of Ocean University of China. A total of 18 rats were randomly divided into three groups. Two groups were administered the GS aqueous solution *via* oral and transdermal routes, respectively. The third group was treated with GS@MT-Men (1:1) DES *via* transdermal administration. Transdermal administration was performed on the dorsal skin of the rats, with a dosage of 50 mg/kg for all groups.

#### 2.9.2 Collection and processing of plasma samples

Plasma samples (0.3 mL) were collected from the rats' orbital vein at fixed time points for the oral administration group (5, 10, 15, 30, 60, 90, 120, 180, 240, 360, 480, 600, 1440 min) and transdermal administration group (10, 20, 30, 45, 60, 120, 240, 360, 480, 600, 1440 min). After the plasma samples collection, all the rats were sacrificed and then uniformly handed over to the environmental protection company for disposal by the laboratory animal center. The collected samples were immediately transferred into heparinized tubes and centrifuged at 14000 rpm and 4 °C for 5 min using a high-speed refrigerated centrifuge (D3023R, DLAB). 50 μL of plasma samples were mixed thoroughly with 500 μL of n-butanol, vortexed for 4 min, and centrifuged again under the same conditions. 450 μL of the supernatant was collected and evaporated using a centrifugal concentrator. The residue was reconstituted with 70 μL of methanol and centrifuged at 14000 rpm and 4 °C for 5 min. Finally, 60 μL of the supernatant was collected for LC-MS/MS analysis.

#### 2.9.3 LC-MS/MS analysis

UPLC analytical conditions: The ACQUITY UPLCRBEH C18 column (1.7 μm, 2.1 × 100 mm) was used for analysis. The aqueous phase (A) in the mobile phase was an aqueous solution containing 3 mM ammonium acetate and 0.1% formic acid, while methanol was selected as the organic phase (B). Flow rate: 0.2 mL/min; injection volume: 5 μL; auto-sampler temperature: 10 °C; column temperature: 40 °C. The specific gradient elution program was shown in the Table 2.

**Table 2:** The ratio of aqueous phase (A) to organic phase (B) at different analysis time intervals.

Time	A%	B%
0.00–0.50 min	30	70
0.50–1.50 min	3	97
1.50–4.50 min	3	97
4.50–5.00 min	30	70

Mass spectrometric conditions: electrospray ionization (ESI) was selected as the ion source; the scanning mode was multiple reaction monitoring (MRM); detection mode was negative ion; the dwell time was 200 ms. The ion pairs monitored for quantitative analysis were: GS: *m*/*z* 433.40 → 225.20, with a collision energy (CE) of 15.0 eV; Taxifolin (internal standard): m/z 302.90 → 124.95, with a CE of 10.25 eV.

#### 2.9.4 Pharmacokinetic parameter calculation

The pharmacokinetic parameters after administration were calculated using the non-compartmental model in WinNonlin 5.3 software (Pharsight, USA). Both the maximum plasma concentration (C_max_) and the peak time (T_max_) were the actual measured values. The area under the curve (AUC_0-t_) was calculated using the trapezoidal method, while AUC_0-∞_ was the sum of AUC_0-t_ and C_t_/k_e_. Where C_t_ represents the last measurable drug concentration, and k_e_ is the elimination rate constant. The elimination half-life (t_1/2_) was calculated as 0.693/k_e_. The mean residence time (MRT) was derived from AUMC/AUC, and plasma clearance (CL) was calculated as D/AUC_0-∞_, where D represents the administered dose.

### 2.10 *In vivo* pharmacodynamic studies of GS

#### 2.10.1 Establishment of the rheumatoid arthritis model

An adjuvant-induced arthritis (AIA) model in rats was established by injecting CFA into the plantar region (Ahmed et al., 2015). Specifically, 0.1 mL of CFA (10 mg/mL) was injected intradermally into the left hind plantar region. The injection was considered successful when visible skin swelling formed at the injection site. The modeling phase lasted for 7 days.

#### 2.10.2 Determination of the treatment regimen

42 RA rats were randomly divided into 7 groups: Control, GS (50 mg/kg), GS (100 mg/kg), MT-Men DES (50 mg/kg), MT-Men DES (100 mg/kg), GS@MT-Men DES (50 mg/kg), GS@MT-Men DES (100 mg/kg). Each group consisted of 6 rats. Except for the control group, the other groups were treated daily. The GS treatment group received an oral solution of GS aqueous solution, while the DES treatment groups were treated with local application of DES at the ankle joint. The total treatment duration was 21 days. During the treatment period, the rats' body weight, paw thickness, and paw volume were recorded every 2 days. After the experiment, blood was collected from the rats' abdominal aorta into evacuated blood collection tubes, left at room temperature for 2 h, and then centrifuged at 4 °C and 2000 ×*g* for 15 min using a high-speed refrigerated centrifuge (PK-20 M, Hunan Pingke Scientific Instrument Co., Ltd). The upper serum was collected and analyzed by ELISA. Finally, the ankle joints were harvested, fixed in 4% paraformaldehyde for 24 h, and subsequently subjected to decalcification, embedding, sectioning, and staining with hematoxylin and eosin for pathological observation. The animals' carcasses were handed over to an environmental protection company for centralized disposal by the laboratory animal center.

### 2.11 Safety assessment

#### 2.11.1 Skin irritation assay

A small amount of blank MT-Men (1:1) DES and GS@MT-Men (1:1) DES samples were locally applied to the dorsal skin of rats for 12, 24, and 48 h, respectively. The skin tissue was then collected and fixed in 4% paraformaldehyde. The fixed tissues were sequentially dehydrated, embedded, sectioned, and stained with hematoxylin and eosin. Finally, the specimens were examined under a high-powered optical microscope (BX53, Olympus Corporation, Japan), with the objective lens set to 4× for observation.

#### 2.11.2 Cytotoxicity assay

HaCaT cells were seeded into 96-well cell culture plates and incubated at 37 °C with 5% CO_2_ for 24 h. Different concentrations of the samples were then added, and incubation continued for an additional 24 h. Afterward, 10 μL of CCK-8 enhanced solution was added to each well, and the plates were incubated for another hour. Absorbance was measured at 450 nm using an xMark microplate spectrophotometer (Bio-Rad, USA). Throughout the experiment, cell culture was maintained in MEM (with NEAA), 15% FBS, and 1% P/S. The formula for calculating cell viability percentage is as follows:

(3) $$\begin{array}{c} \mathrm{Cell viability}\;\left(\%\right)=\left[\frac{\mathrm{Asample}-\mathrm{Ablank}}{\mathrm{Acontrol}-\mathrm{Ablank}}\right]\times 100\% \end{array}$$

Where *A*_*sample*_ is the absorbance of the wells containing both cells and samples, while *A*_*control*_ and *A*_*blank*_ represent the absorbance of the wells containing only cells and the wells without cells, respectively.

### 2.12 Statistical analysis

Statistical analysis was performed using Origin 2021 software. The results are presented as mean ± standard deviation. Differences between two groups were analyzed using Student's *t*-test, while differences among more than two groups were assessed using analysis of variance (ANOVA). A *p-*value <0.05 was considered statistically significant.

## 3 Results and discussion

### 3.1 Formation and characterization of MT-Men DES

**Fig. 1:**
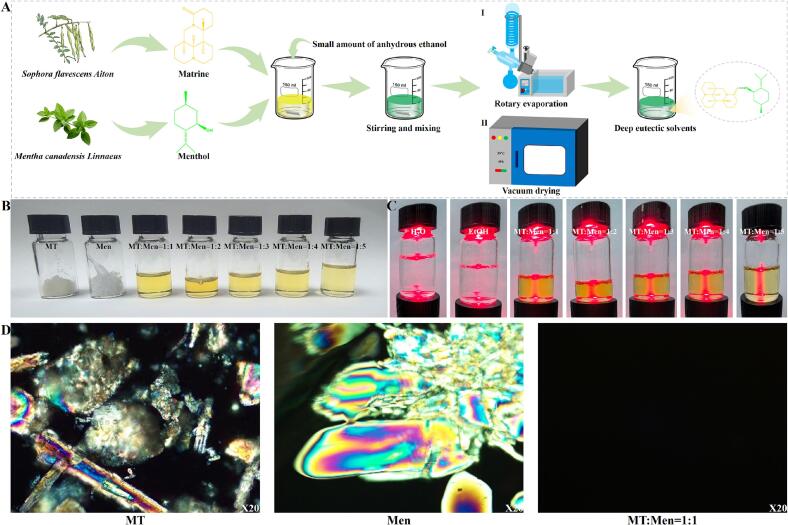
Preparation and characterization of MT-Men DES. (A) Schematic diagram of the MT-Men DES preparation process; (B) Visual observation of MT-Men DES with different molar ratios and pure components; (C) The Tyndall effect of MT-Men DES; (D) Observation of MT-Men (1:1) DES and pure component under a dark field microscope.

MT-Men DES were obtained by mixing the natural small molecules MT and Men, followed by drying and equilibration. The specific preparation process is shown in Fig. 1A. We first obtained a stable liquid eutectic mixture by mixing MT and Men in an equimolar ratio of 1:1. Based on the concept of binary phase behavior, we continued to increase the amount of Men and successfully obtained MT-Men DES systems with ratios of 1:2 to 1:5. Compared to MT and Men, the prepared MT-Men DES appeared as a uniform, pale yellow transparent liquid (Fig. 1B). To distinguish the prepared MT-Men DES samples from conventional solvents (such as water, ethanol, *etc.*), we used a laser pointer to separately irradiate water, ethanol, and the prepared DES systems. The results showed that, compared to ethanol and water, the DES samples exhibited a significant Tyndall effect (Fig. 1C), indicating that DES exhibited colloidal properties. Further observation using POM revealed that, compared with MT and Men, MT-Men (1:1) DES exhibited typical amorphous characteristics (Fig. 1D). The POM results of MT-Men (1:2–5) were exhibited in the supplementary materials (Fig. S1–4).

**Fig. 2:**
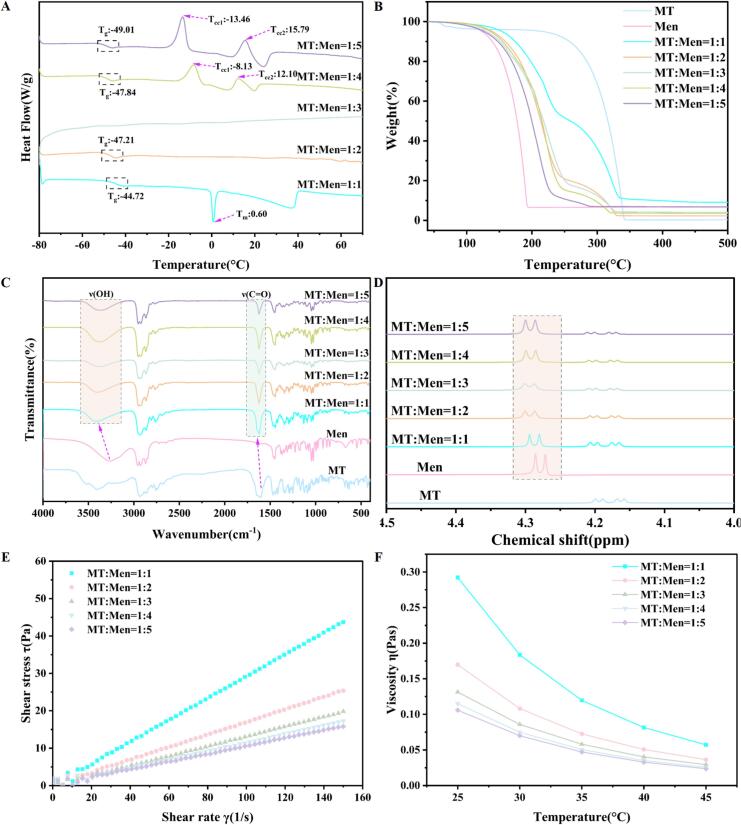
Thermodynamic properties and formation mechanisms of MT-Men DES. (A) The DSC curve of MT-Men DES; (B) The TG analysis of MT-Men DES and pure components; (C) The FTIR analysis of MT-Men DES and pure components; (D) ^1^H NMR spectra (400 MHz, DMSO‑*d*_6_) of MT-Men DES and pure components in the range of 4.0–4.5 ppm; (E) Shear stress of MT-Men DES as a function of shear rate at 25 °C; (F) Viscosity of MT-Men DES as a function of temperature.

To further illustrate the amorphous state of the MT-Men DES, DSC is used to determine the thermal transitions. MT exhibited a single endothermic peak at 61.85 °C, corresponding to its melting point, while Men exhibited endothermic peaks at 45.92 °C and 216.60 °C, corresponding to its melting point and boiling point, respectively (Fig. S5). Compared with MT and Men, the glass transition temperature (T_g_) range of MT-Men DES was observed to be between −44 and − 50 °C (Fig. 2A). In addition, we also observed that MT-Men (1:4) DES and MT-Men (1:5) DES exhibited an exothermic peak at low temperatures, which was a typical cold crystallization phenomenon (Wunderlich, 1958). This is the crystallization process that amorphous materials undergo when reheated above the glass transition temperature (Manley, 1989). A similar phenomenon was also observed in studies of choline chloride (ChCl)-sucrose (4:1) DES and ChCl-xylose (3:1) DES systems (Craveiro et al., 2016). Additionally, no endothermic peak for MT and Men was observed in the thermograms of all the prepared DES. These results indicate the conversion of MT and Men from a crystalline to an amorphous form.

TG is used to evaluate the thermal behavior of MT-Men DES and their pure components. As shown in Fig. 2B, the cumulative weight loss percentage of MT and Men reached 99% and 93%, respectively. Compared to MT and Men, the cumulative weight loss percentage of all the prepared MT-Men DES showed varying degrees of change. Among them, the MT-Men (1:1) DES exhibited optimal thermal stability, with a cumulative weight loss percentage of only 91%, indicating its excellent thermodynamic stability. To understand the formation mechanism of MT-Men DES, the weak interaction between MT and Men can be analyzed by examining the vibrational changes of characteristic functional groups in the FTIR profiles. As shown in Fig. 2C, the MT-Men DES spectrum was compared to the spectra of the pure components, where the variation of characteristic peaks linked to specific functional groups can be clearly observed, such as the stretching vibration changes of the ketone carbonyl (-C=O) and hydroxyl (-OH) groups. For Men, a typical broad characteristic absorption peak was observed at 3260 cm^−1^, corresponding to -OH stretching vibrations. A remarkable blue shift from 3260 cm^−1^ to (3410–3361 cm^−1^) for MT-Men (1:1–5) DES was exhibited after forming the DES, which was associated with possible hydrogen bond formation. For MT, the ketone carbonyl (-C=O) stretching vibration manifested as an absorption peak at 1604 cm^−1^. After forming MT-Men DES, this peak shifted to higher wavenumbers. In addition, we also observed that after the formation of the MT-Men DES, the intensity of the absorption peak of the hydroxyl (-OH) group in Men showed varying degrees of enhancement, and the peak shape exhibited broadening and attenuation. These results indicate that the formation of DES might be promoted by hydrogen bonding between the MT (-C=O) and Men (-OH).

To further demonstrate that hydrogen bonds facilitate the formation of MT-Men DES, ^1^H NMR studies are conducted to evaluate the interactions between MT and Men in the MT-Men DES. As shown in Fig. 2D, the proton signal of the hydroxyl (-OH) group in Men (δ_H-8_ = 4.279 ppm) was used as a reference. Proton signal peak of the hydroxyl (-OH) group in Men shifted to the range of 4.287–4.294 ppm after participating in the formation of MT-Men DES. In addition, the proton signal peak of the Men hydroxyl (-OH) group exhibited a flattened and broadened shape. This is similar to the changes in the hydroxyl proton observed in the ChCl- acetic acid (Aa) DES system as reported previously (Cheng et al., 2024). These results further corroborate the FTIR findings, indicating that hydrogen bonds were formed between MT and Men. The complete ^1^H NMR spectra of MT-Men DES and pure components, along with the analysis results of all proton signals, were shown in Fig. S6 and Table S1–6, respectively. The chemical structures of MT and Men, along with their atomic numbers, were shown in Fig. S7.

Rheological behavior is an important characteristic of fluids, influencing viscosity dependent applications such as pumping, mass transfer rates, and hydrodynamics (Elhamarnah et al., 2019). Temperature is one of the key factors affecting fluid viscosity. Therefore; it is necessary to evaluate the rheological behavior of MT-Men (1:1–5) DES. As shown in Fig. 2E; the ratio of shear stress to shear rate of the MT-Men (1:1–5) DES exhibited linear relationship under the temperature conditions of 25 °C; which confirms to the characteristics of typical Newtonian fluids. As the temperature gradually increased to 30; 35; 40; and 45 °C; this linear relationship still remained unchanged (Fig. S8–11). This indicates that MT-Men (1:1–5) DES have good thermodynamic stability. The viscosity variation with temperature was measured within the same temperature range. It can be seen that the viscosities of MT-Men (1:1–5) DES gradually decreased with increasing temperature (Fig. 2F). Among these; MT-Men (1:1) DES exhibited more significant changes. The main reason is the disruption of some hydrogen bonds increases the molecular mobility (Omar and Sadeghi, 2022). Besides; higher temperatures reduce the internal resistance of the mixed system; leading to an increase in molecular fluidity (Chemat et al., 2016; Ghaedi et al., 2017). It was worth noting that the viscosity of MT-Men DES did not increase with the increase in the molar ratio of Men but instead gradually decreased under the same temperature conditions. This is primarily because excess Men tends to disrupt the pairing and dynamic protonation process between MT and Men, ultimately reducing the viscosity of DES. This has been confirmed in studies of choline and geranic acid (CAGE) systems with different molar ratios (Tanner et al., 2018).

### 3.2 MD simulations and DFT calculations

**Fig. 3:**
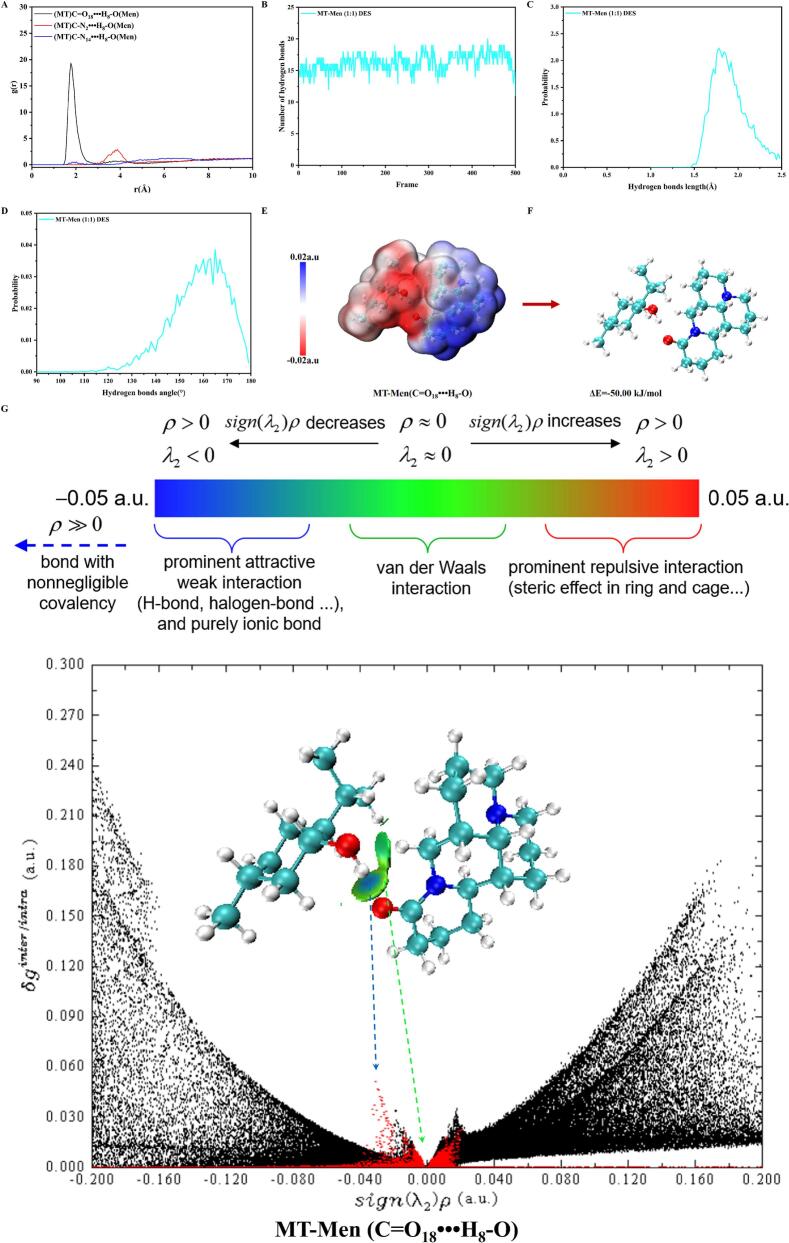
MD simulations and DFT calculations of the MT-Men DES. (A) RDF analysis between specific atoms in MT-Men DES; (B) Average number of hydrogen bonds in MT-Men DES. The probability distribution of the angle (C) and length (D) of hydrogen bonds in MT-Men DES; (E) The corresponding molecular electrostatic potential (ESP) diagram of MT-Men molecular pairs (red: negatively charged; blue: positively charged); (F) Calculations of the binding energy between MT and Men in MT-Men DES; (G) Visualization of non-covalent bonds between MT and Men based on the independent gradient model using Hirshfeld partition (IGMH) analysis and scatter plot of MT-Men molecular pairs. (For interpretation of the references to colour in this figure legend, the reader is referred to the web version of this article.)

The use of MD simulations can further demonstrate the hydrogen bonding sites between the components of DES through theoretical calculations. According to the previously defined geometric criteria for hydrogen bond existence, a distance of less than 3.5 Å between the donor hydrogen and the acceptor is considered indicative of a hydrogen bond. Specifically, a distance of less than 2.5 Å is characteristic of a strong hydrogen bond (Jeffrey and Jeffrey, 1997). Therefore, we calculated the g(r) values between the nitrogen or oxygen atoms in MT and the hydroxyl protons of Men (Fig. 3A). The results indicated that the hydrogen bonds in the MT-Men DES were primarily formed between the oxygen atoms of the carbonyl groups (-C=O_18_) in MT and the hydroxyl protons (-OH_8_) in Men. Further calculations revealed that the average number of hydrogen bonds is 16.32, with the bond angle and bond length of the hydrogen bonds being approximately 165° and 1.78 Å, respectively (Fig. 3BCD).

DFT calculations further elucidate the formation mechanism of MT-Men DES at the molecular level. As shown in Fig. 3E, the electrostatic potential (ESP) diagram reflects the magnitude of the electrostatic potential on the molecular surface. Based on the constructed model, it can be observed that, during the binding process of MT and Men, the positively charged region of MT was electrostatically attracted to the negatively charged region of Men. The interaction energy was further quantitatively calculated, revealing that the interaction energy between MT and Men was −50 kJ/mol (Fig. 3F). IGMH analysis further visualized the non-covalent interactions between MT and Men (Fig. 3G). The results indicate that, in addition to hydrogen bonds, van der Waals forces also play a significant role in driving the binding between MT and Men.

### 3.3 Study on the *in vitro* permeability of GS

MT-Men (1:1–5) DES were selected for the determination of the saturated solubility of GS. As shown in Fig. S12, the solubility of GS in MT-Men (1:1) DES reached 65.69 ± 6.12 mg/mL. When the molar ratio of MT to Men reached 1:2 and 1:3, the solubility of GS rapidly decreased to 22.34 ± 6.02 mg/mL and 15.82 ± 2.03 mg/mL, respectively. However, as the amount of Men continued to increase, the solubility of GS tended to stabilize, reaching 6.62 ± 0.18 mg/mL and 5.43 ± 0.39 mg/mL in the MT-Men (1:4) DES and MT-Men (1:5) DES, respectively. This is mainly because MT is hydrophilic, while Men is hydrophobic. When the proportion of MT in MT-Men DES increases, there is better compatibility with GS. However, as the proportion of Men increases, MT-Men DES tends to exhibit more hydrophobic properties, weakening the dissolution performance of GS. Similar conclusions were drawn in the study of CAGE systems with different molar ratios (Tanner et al., 2018). In addition; the difference in solubility of GS might also be associated with intermolecular interactions. Generally; stronger intermolecular interactions lead to greater solubility (Huber et al., 2022). A previous study showed that the DES formed by choline chloride with ascorbic acid or propylene glycol can effectively enhance the solubility of dapsone; with hydrogen bonding being the primary driving force (Trombino et al., 2022).

**Fig. 4:**
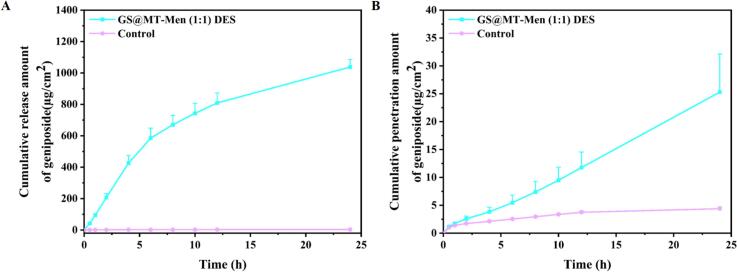
Study on the *in vitro* release and permeability of GS@MT-Men (1:1) DES. (A) *In vitro* release test of GS@MT-Men (1:1) DES; (B) *In vitro* permeation test of GS@MT-Men (1:1) DES.

*In vitro* release test is one of the most important tools in drug development and the approval process for semi-solid formulations (Shah et al., 2022). It primarily involves simulating the release environment of the drug *in vitro* to evaluate the rate and extent of drug release after topical administration; thereby assessing the performance of the topical formulation. This is crucial for quality control of the formulation. Therefore; it is necessary to evaluate the release behavior of GS in MT-Men (1:1) DES. As shown in Fig. 4A; MT-Men (1:1) DES exhibited rapid release; with a cumulative release amount of 1037.80 ± 47.58 μg/cm^2^ within 24 h; while the GS aqueous solution showed almost no release; with a 24-h cumulative release amount of only 3.43 ± 0.18 μg/cm^2^. To gain a deeper understanding of the mechanism of GS release from MT-Men (1:1) DES and water; four mathematical models; including zero-order; first-order; Higuchi; and Ritger-Peppas equations; were used to fit the *in vitro* release curve of GS (See Table 3). For MT-Men (1:1) DES; the best fit was obtained by applying the first-order release equation; with the correlation coefficient (R^2^) being 0.9958. This indicates that GS in MT-Men (1:1) DES exhibits a sustained-release mechanism; where GS is released slowly and non-constantly. In contrast; GS in water follows the Ritger-Peppas release kinetics. Notably; the different values of *n* in the Ritger-Peppas equation represent different release mechanisms (Sun et al., 2014). An *n* value <0.45 indicates Fick's diffusion, while an *n* value >0.89 indicates a dissolution-controlled mechanism. If *n* is between 0.45 and 0.89, it suggests a combined effect of Fick's diffusion and dissolution. Therefore, GS in water exhibits Fick's diffusion.

**Table 3:** Different model fitting results for the *in vitro* release test of GS in DES and water.

	Zero-order release equation	First-order release equation	Higuchi equation	Ritger-Peppas equation
GS@MT-Men (1:1) DES	Q = 42.63 t + 192.81; R^2^ = 0.8142	Q = 1098.61(1-e^-0.12t^); R^2^ = 0.9958	Q = 254.97 t^1/2^–108.59; R^2^ = 0.9661	Q = 190.68 t^0.56^; R^2^ = 0.9479
GS aqueous solution	Q = 0.11 t + 1.33; R^2^ = 0.8315	Q = 3.09(1-e^-0.26t^); R^2^ = 0.8084	Q = 0.63 t^1/2^ + 0.59; R^2^ = 0.9674	Q = 1.19 t^0.35^; R^2^ = 0.9813

The *in vitro* permeation test was designed to evaluate whether MT-Men (1:1) DES can assist GS in passing through the skin barrier and entering the subcutaneous tissue. As shown in Fig. 4B, after applying GS@MT-Men (1:1) DES and GS aqueous solution to the stratum corneum layer of porcine skin for 24 h, only the MT-Men (1:1) DES exhibited sustained delivery, with a cumulative penetration amount per unit area reaching up to 25.29 ± 6.83 μg/cm^2^. In contrast, for the GS aqueous solution, most of the GS was retained on the skin surface, with only a minor fraction reaching the receptor cell and being detected. The cumulative penetration amount per unit area was only 4.38 ± 0.32 μg/cm^2^.

To further illustrate the difference in the penetration effect of MT-Men (1:1) DES and water, a linear fit was performed for the 0–12 h interval. The detailed calculation results are shown in Table 4. Compared to water, the ER value of MT-Men (1:1) DES reached 4.05, indicating better permeation-promoting effects. Since MT-Men (1:1) DES does not immediately achieve stable and sustained penetration after application to the skin and requires a certain lag time, the T_lag_ value was also calculated to better evaluate the time required for GS to reach distribution equilibrium in the skin (Nugroho et al., 2004). Notably, MT-Men (1:1) DES showed a shorter T_lag_ value, indicating that this system can enhance the absorption and distribution efficiency of GS. The 24-h skin retention analysis indicated that MT-Men (1:1) DES can assist GS in penetrating the stratum corneum barrier to reach deeper layers of the skin, with a 24-h cumulative retention amount reaching 12.40 ± 0.30 μg/cm^2^, which is 2.8-fold higher than that of the GS aqueous solution group (Fig. S13). To sum up, these results indicate that MT-Men (1:1) DES can facilitate the transdermal delivery of GS.

**Table 4:** Summary of the calculation results for key parameters in the *in vitro* permeation test of GS@MT-Men (1:1) DES and GS aqueous solution.

	J_ss_(μg/cm^2^/h)	T_lag_(h)	ER	Q_24h_(μg/cm^2^)
GS@MT-Men (1:1) DES	0.89 ± 0.0320	0.58 ± 0.2161	4.05	25.29 ± 6.83
GS aqueous solution	0.22 ± 0.0111	1.14 ± 0.0751	1.00	4.38 ± 0.32

**Fig. 5:**
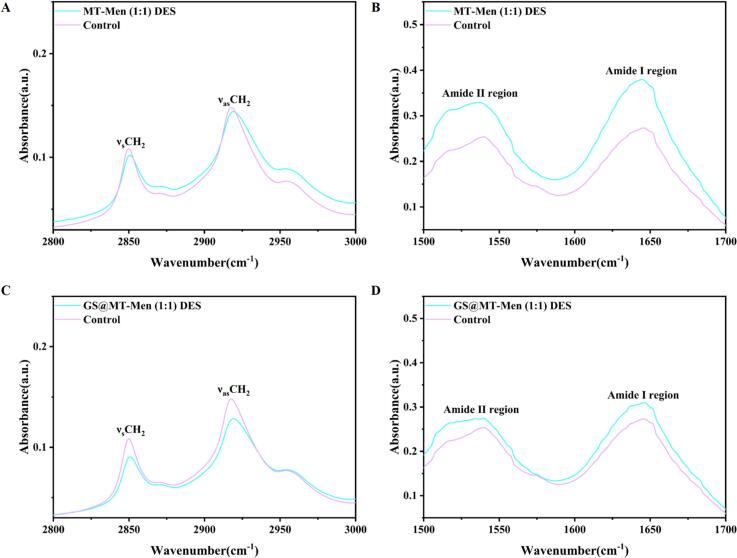
Study on the transdermal mechanism of GS@MT-Men (1:1) DES. ATR-FTIR spectra of the stratum corneum of porcine skin after treatment with MT-Men (1:1) DES were shown in the lipid regions (A) and amide band regions (B); After treatment with GS@MT-Men (1:1) DES, the spectra were shown in the lipid regions (C) and amide band regions (D). Untreated skin was used as the control group (the concentration of GS was 10 mg/mL).

ATR-FTIR has been widely used to study the properties of the stratum corneum structure (Brancaleon et al., 2001). To further investigate the permeation mechanism of MT-Men (1:1) DES; ATR-FTIR spectroscopy was employed to gain insights into the effect of MT-Men (1:1) DES on the structure of the skin's stratum corneum. As shown in Fig. 5AB; the FTIR spectra of the skin treated with MT-Men (1:1) DES and untreated skin were first determined. In the lipid region (2800–3000 cm^−1^); for untreated skin; the lipids exhibited characteristic symmetrical and asymmetrical vibrational absorption peaks at 2849.89 cm^−1^ (ν_s_CH_2_) and 2917.71 cm^−1^ (ν_as_CH_2_); which is consistent with previous research (Wang et al., 2022). Compared to untreated skin; both ν_s_CH_2_ and ν_as_CH_2_ in the MT-Men (1:1) DES-treated group shifted toward higher wavenumbers. Previous studies have shown that the displacement of CH_2_ stretching vibration peak reflects the degree of freedom of the lipid side chains (Rerek et al., 2005). Typically; the CH_2_ vibration absorption peak shifts to higher wavenumbers; indicating that CH_2_ is deflected; leading to a disruption of the ordered arrangement of lipid structures and a transition to a disordered state; ultimately promoting drug absorption (Mendelsohn et al., 2006; Yang et al., 2021). Additionally, the FTIR spectra of the stratum corneum treated with MT-Men (1:1) DES exhibited a significant reduction in the peak height of CH_2_, which is a typical characteristic of lipid extraction. According to previous studies, lipid extraction is associated with enhanced permeation (Karande et al., 2005). This suggests that the permeation mechanism of MT-Men (1:1) DES primarily involves lipid extraction, accompanied by a degree of disorder in the lipid structure.

Changes in the protein secondary structure were also observed in the amide band region (1500–1700 cm^−1^), particularly represented by the amide I band. For untreated skin, a typical characteristic absorption peak of the amide I band was observed at 1645.43 cm^−1^. Compared to untreated skin, the skin treated with MT-Men (1:1) DES showed a shift of the amide I band toward lower wavenumbers, which may be related to the transition of keratin conformation from α-helix to β-fold (Li et al., 2022). The deformation of keratin arrangement leads to an increase in intermolecular spacing (Ibrahim and Li, 2009). Additionally, FTIR spectra of the skin treated with GS@MT-Men (1:1) DES were also collected (Fig. 5CD). Obviously, the addition of GS did not enhance the displacement of CH_2_, indicating that it does not promote the disorder of the lipid structure. However, it was observed that in the lipid band region, the CH_2_ peak height of the lipid band treated with GS@MT-Men (1:1) DES decreased by nearly 7% compared to MT-Men (1:1) DES, exhibiting a stronger lipid extraction effect. Moreover, the skin treated with GS@MT-Men (1:1) DES showed a shift to higher wavenumbers in the amide I band. These results suggest that the addition of GS helps enhance the lipid extraction effect of MT-Men (1:1) DES, thereby weakening the barrier of the stratum corneum and ultimately facilitating the delivery of GS.

To further determine the specific distribution of MT-Men (1:1) DES-assisted GS after passing through the skin stratum corneum, cy3 (a commonly used anthocyanin dye) was employed to fluorescently label GS. As shown in Fig. S14, the cy3-GS@MT-Men (1:1) DES-treated skin showed high fluorescence intensity in the stratum corneum, epidermis, and dermis, indicating that it can effectively deliver GS through the stratum corneum into the dermis. However, the skin samples treated with the cy3-GS aqueous solution showed only weak fluorescence in the dermis, with almost no detectable fluorescence in the subdermal layer, and the majority of the GS accumulated in the stratum corneum (Fig. S15). In addition, we also observed that the penetration of MT-Men (1:1) DES-assisted GS through the skin was uniform, with no selective transport through hair follicles. Therefore, the MT-Men (1:1) DES we prepared appears to uniformly enhance stratum corneum permeability, rather than facilitating localized transport through pathways like hair follicles or pores, as seen in electrophoresis and sonophoresis (Denet et al., 2004; Kushner IV et al., 2008). This is consistent with the conclusions drawn from exploring the mechanism of the CAGE system for transdermal delivery of insulin (Qi and Mitragotri, 2019).

### 3.4 *In vivo* transdermal delivery of GS

#### 3.4.1 *In vivo* pharmacokinetic evaluation

**Fig. 6:**
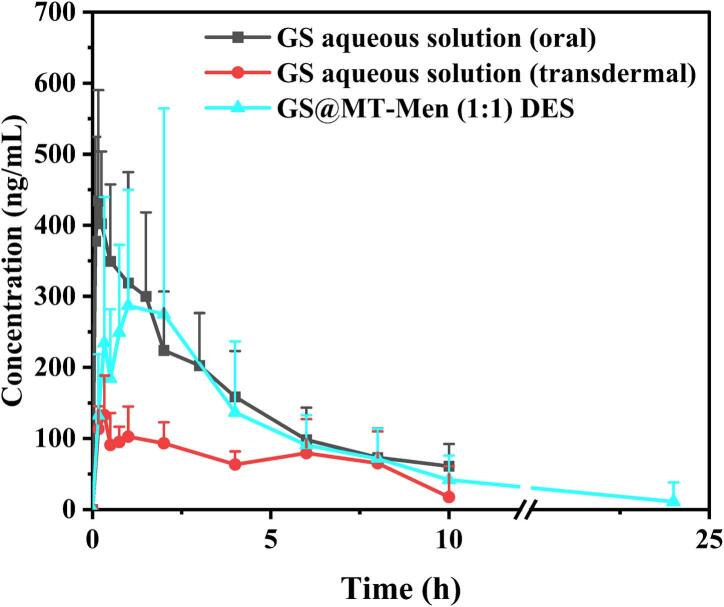
Mean plasma concentration of GS as a function of time. (black: oral administration of GS aqueous solution, red: transdermal administration of GS aqueous solution, cyan: transdermal administration of GS@MT-Men (1:1) DES, *n* = 6). (For interpretation of the references to colour in this figure legend, the reader is referred to the web version of this article.)

Based on the results of the *in vitro* release and permeation tests, the MT-Men (1:1) DES was selected for the *in vivo* pharmacokinetic study to assess the bioavailability of GS. The mean plasma concentration-time curves for the GS aqueous solution and the GS@MT-Men (1:1) DES are shown in Fig. 6. Among the rest, the GS aqueous solution was chosen as the control, with two administration routes: transdermal and oral. Plasma concentrations of GS following transdermal administration of the GS@MT-Men (1:1) DES were significantly higher than those of the GS aqueous solution administered *via* the same route. Furthermore, compared to the oral administration of the GS aqueous solution, the GS@MT-Men (1:1) DES transdermal group showed detectable levels of GS for up to 24 h. The main pharmacokinetic parameters were calculated and listed in Table 5.

**Table 5:** Pharmacokinetic parameters for oral and transdermal administration.

	Oral administration	Transdermal administration	
	GS aqueous solution	GS aqueous solution	GS@MT-Men (1:1) DES
*C*_max_ (ng/mL)	501 ± 124	152 ± 35.9	437 ± 260
*T*_max_ (h)	0.36 ± 0.34	2.25 ± 2.91	0.88 ± 0.65
*t*_1/2_ (h)	3.21 ± 1.80	3.58 ± 0.72	5.24 ± 2.38
AUC_0-t_ (h·ng/mL)	1581 ± 463	665 ± 231	1543 ± 949
AUC_0-∞_ (h·ng/mL)	1861 ± 602	945 ± 188	1826 ± 891
CL (mL/h·Kg)	28,692 ± 9746	49,954 ± 10,688	34,860 ± 28,491
MRT (h)	3.36 ± 0.78	3.74 ± 1.11	4.31 ± 2.71
F_r_ (%)	100.00	42.06	97.60

After oral administration of the GS aqueous solution, GS was rapidly absorbed, reaching a C_max_ of 501 ± 124 ng/mL at 0.36 ± 0.34 h, and was then completely eliminated within 10 h. However, when administered transdermally, the T_max_ of GS in the GS aqueous solution was significantly delayed to 2.25 ± 2.91 h, with a lower C_max_ (152 ± 35.9 ng/mL) observed. Compared to the GS aqueous solution transdermal administration group, the GS@MT-Men (1:1) DES transdermal administration group showed a significantly improvement in pharmacokinetic behavior, with a higher C_max_ (437 ± 260 ng/mL) and a lower T_max_ (0.88 ± 0.65 h). Additionally, the AUC values were substantially increased. The relative bioavailability (F_r_) of GS increased from 42.06% to 97.60%. Furthermore, the t_1/2_ of the GS aqueous solution was similar after both transdermal and oral administration, while the GS@MT-Men (1:1) DES significantly prolonged the t_1/2_, allowing GS to exert its therapeutic effects more durably. In summary, these results indicate that the GS@MT-Men (1:1) DES can effectively enhance the transdermal penetration of GS, enabling continuous delivery for up to 24 h, which aligns with the findings from the *in vitro* permeation test.

#### 3.4.2 *In vivo* pharmacodynamic evaluation

**Fig. 7:**
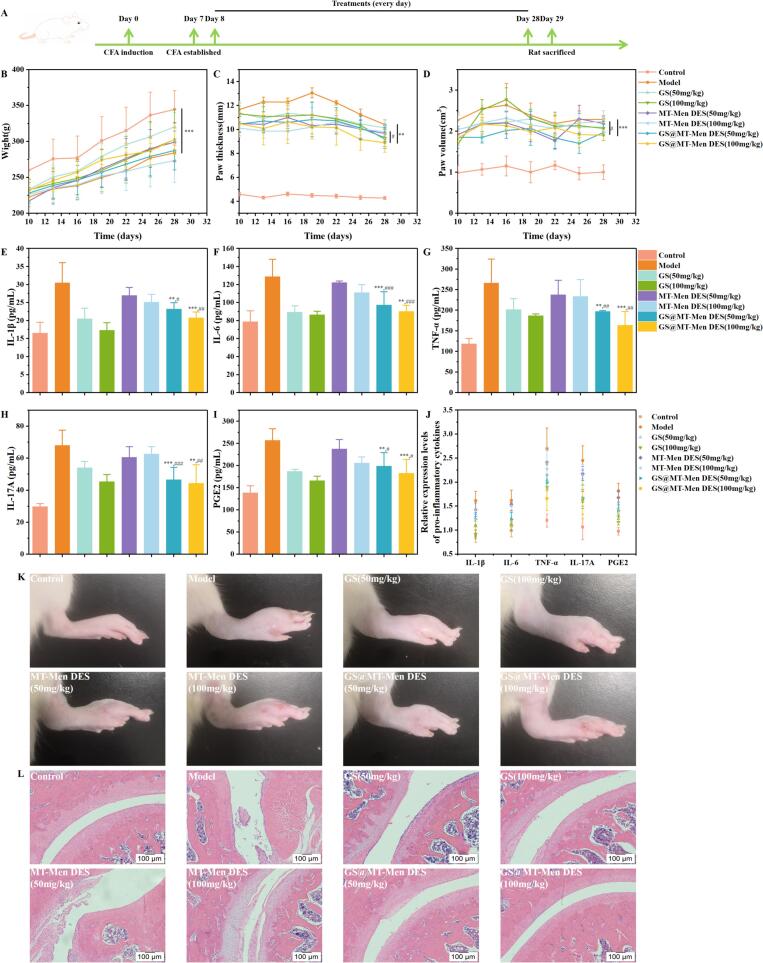
GS@MT-Men (1:1) DES effectively alleviates inflammation in RA rats. (A) Establishment of RA rats and a schematic representation of their treatment scheme; (B—D) Measurement of body weight, paw thickness, and paw volume in rats; (E-I) Quantification of pro-inflammatory cytokines in the serum of rats measured by ELISA; (J) Relative expression levels of pro-inflammatory cytokines in rat serum; (K) Visual observation of the left paw of rats on day 28; (L) Histopathological analysis of H&E-stained sections of the rat ankle joint. **: *p* < 0.01, ***: *p* < 0.001, compared with the model group; #: *p* < 0.05, ##: *p* < 0.01, ###: *p* < 0.001 compared with the MT-Men (1:1) DES group.

*In vivo* pharmacokinetic studies have shown that GS@MT-Men (1:1) DES can enhance the transdermal bioavailability of GS. Therefore, in order to further demonstrate the effectiveness of GS@MT-Men (1:1) DES in disease treatment, an RA model was constructed to evaluate the anti-inflammatory effect of GS@MT-Men (1:1) DES. Given that no marketed products containing GS are available, the GS aqueous solution oral administration group was selected as the reference. The treatment cycles for both oral and transdermal administration are shown in Fig. 7A**.** The body weight of the rats was monitored, and compared to the control group, the RA rats exhibited a significantly slower growth rate (Fig. 7B). Additionally, the thickness and volume of the rats' paws were measured (Fig. 7C and D). During the model establishment phase, the paw thickness of the RA rats increased from 4.29 mm to 10.96 mm, and the paw volume increased from 1.15 cm^3^ to 2.22 cm^3^. Following the local application of GS@MT-Men (1:1) DES, paw swelling was significantly inhibited. Notably, when the dosage was 100 mg/kg, the paw thickness and paw volume decreased to 8.88 mm and 1.90 cm^3^, respectively.

An ELISA kit was used to quantify the levels of inflammatory factors (IL-1β, IL-6, TNF-α, IL-17 A, PGE2) in the serum of rats. As shown in Fig. 7E-I, compared to the control group, the levels of inflammatory factors in the model group were significantly increased, with 1.85-fold (IL-1β), 1.63-fold (IL-6), 2.25-fold (TNF-α), 2.29-fold (IL-17 A), and 1.86-fold (PGE2). After treatment with oral GS aqueous solution and transdermal GS@MT-Men (1:1) DES, the serum levels of inflammatory factors in RA rats significantly decreased. Furthermore, this decrease was notably dose-dependent.

Compared to the GS aqueous solution oral administration group, the GS@MT-Men (1:1) DES transdermal treatment group demonstrated a more significant effect in inhibiting the production of TNF-α and IL-17 A inflammatory factors. Further analysis of the relative expression levels of pro-inflammatory factors revealed that TNF-α and IL-17 A had the highest relative expression levels (Fig. 7J). Therefore, we conclude that the key to the effectiveness of GS@MT-Men (1:1) DES in delivering GS for RA treatment lies in its inhibition of the high expression of TNF-α and IL-17 A. Furthermore, a significant reduction in paw swelling was observed in the GS@MT-Men (1:1) DES treatment group of rats (Fig. 7K). H&E staining of ankle joint sections was then performed to assess the histopathological characteristics of the tissue (Fig. 7L). The results showed that, compared to the control group, the model group exhibited significant infiltration of inflammatory cells, synovial hyperplasia, and cartilage erosion, which were consistent with the pathological features of arthritis. After oral administration of the GS aqueous solution and transdermal administration of GS@MT-Men (1:1) DES, inflammation was alleviated to varying degrees in a dose-dependent manner. The transdermal delivery of GS@MT-Men (1:1) DES produced the most significant recovery. These results suggest that the GS@MT-Men (1:1) DES possesses great anti-inflammatory properties.

### 3.5 Safety evaluation

**Fig. 8:**
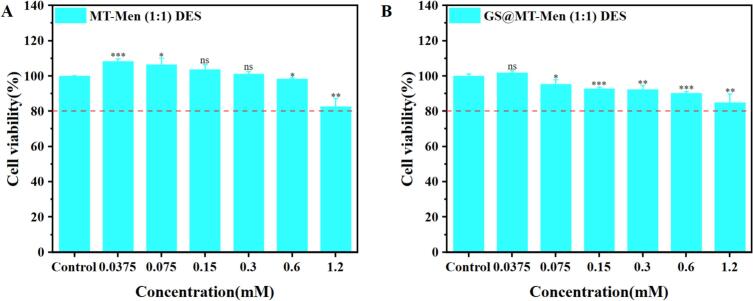
Evaluation of cytotoxicity assay. Cell viability of HaCaT cells treated with different concentrations of (A) MT-Men (1:1) DES and (B) GS@MT-Men (1:1) DES (*n* = 3).

**Fig. 9:**
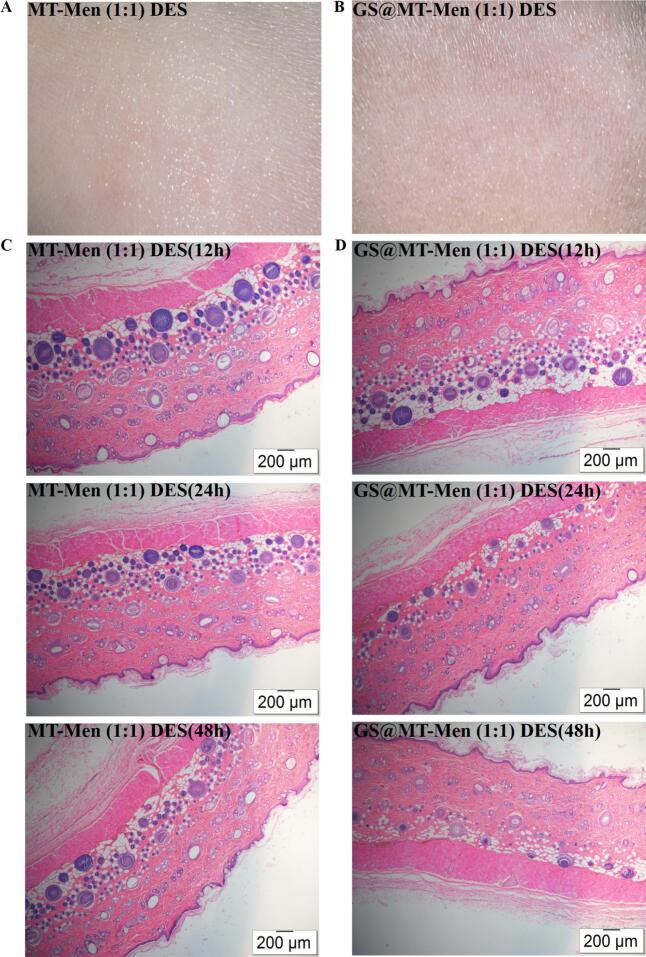
Evaluation of skin irritation test. Visual observation of the skin after treatment with MT-Men (1:1) DES (A) and GS@MT-Men (1:1) DES (B); H&E examinations of the skin sections after treatment with MT-Men (1:1) DES (C) and GS@MT-Men (1:1) DES (D) for 12, 24, and 48 h (*n* = 6).

To assess the safety of MT-Men (1:1) DES for transdermal drug delivery, cytotoxicity tests were performed using HaCaT cells. As shown in Fig. 8AB, CCK-8 assay results indicated that neither MT-Men (1:1) DES nor GS@MT-Men (1:1) DES exhibited significant cytotoxicity within the concentration range of 1.2 mM. A skin irritation test was conducted by applying a small amount of MT-Men (1:1) DES and GS@MT-Men (1:1) DES to the skin on the back of rats, with no allergic reactions or visible inflammation observed (Fig. 9AB). H&E examination of skin sections further revealed no infiltration of inflammatory cells or tissue necrosis at 12, 24, and 48 h (Fig. 9CD). The results for the control group are shown in Fig. S16. In summary, both MT-Men (1:1) DES and GS@MT-Men (1:1) DES can be safely applied to the skin.

## 4 Conclusion

This study focused on developing a novel transdermal penetration enhancer, MT-Men DES, formed by MT from *sophora flavescens* and Men from *Mentha canadensis*. Theoretical calculations and various experimental techniques revealed that the formation of MT-Men DES was primarily driven by weak intermolecular interactions, mainly hydrogen bonds, with van der Waals forces playing a secondary role. *In vitro* release and *In vitro* permeation tests demonstrated that MT-Men (1:1) DES significantly enhanced the transdermal penetration of GS. *In vivo* pharmacokinetic experiments further showed that MT-Men (1:1) DES significantly improved the transdermal bioavailability of GS and could extend its duration of action up to 24 h. This facilitates the sustained penetration and accumulation of GS. Further anti-inflammatory activity assessments revealed that GS@MT-Men (1:1) DES exhibited more significant effects in inhibiting the high expression of inflammatory factors TNF-α and IL-17 A. Finally, safety assessments indicated that GS@MT-Men (1:1) DES has excellent biocompatibility with the skin. In conclusion, this study might provide new references and ideas for addressing the transdermal delivery of hydrophilic drugs.

## CRediT authorship contribution statement

**Hongdou He:** Writing – review & editing, Writing – original draft, Visualization, Formal analysis, Data curation, Conceptualization. **Xinyu Huang:** Formal analysis, Data curation. **Yi Hong:** Writing – review & editing, Supervision, Data curation. **Zhenpeng Qiu:** Formal analysis, Conceptualization. **Fei Xu:** Investigation, Data curation. **Shan Lu:** Writing – review & editing, Validation, Supervision, Project administration, Funding acquisition. **Yujie Guo:** Writing – review & editing, Supervision, Project administration, Funding acquisition, Conceptualization.

## Declaration of competing interest

There are no conflicts of interest to declare.

## Data Availability

Data will be made available on request.
