# Supplementary material for: Deep eutectic solvents based on matrine and menthol: A novel and safe transdermal penetration enhancer
Source: Int J Pharm X. 2026 Apr 3;11:100535. doi: 10.1016/j.ijpx.2026.100535 (PMC13090524; doi:10.1016/j.ijpx.2026.100535)
Supplement: Supplementary file 1 — Supplementary material [file mmc1.docx]

**Deep eutectic solvents based on matrine and menthol: A novel and safe transdermal penetration enhancer**Hongdou He^a, c^, Xinyu Huang^d^, Yi Hong^a, b^, Zhenpeng Qiu^b, d, e^, Fei Xu^c^, Shan Lu^a,^ ^b *^, Yujie Guo^a, b *^

^a^ Research Center for Pharmaceutical Preparations, School of Pharmacy, Hubei University of Chinese Medicine, Wuhan 430065, China

^b^ Hubei Shizhen Laboratory, Wuhan 430061, China

^c^ Institute of Feed Research, Chinese Academy of Agricultural Sciences, Beijing 100081, China

^d^ Hubei Key Laboratory of Resources and Chemistry of Chinese Medicine, School of Pharmacy, Hubei University of Chinese Medicine, Wuhan 430065, China

^e^ Center of Traditional Chinese Medicine Modernization for Liver Diseases, Hubei University of Chinese Medicine, Wuhan, 430065, China

^*^ Corresponding authors at: School of Pharmacy, Hubei University of Chinese Medicine, Wuhan 430065, PR China.

E-mail addresses: lushan9805@163.com (S. Lu), guoyujie@hbucm.edu.cn (Y. Guo)


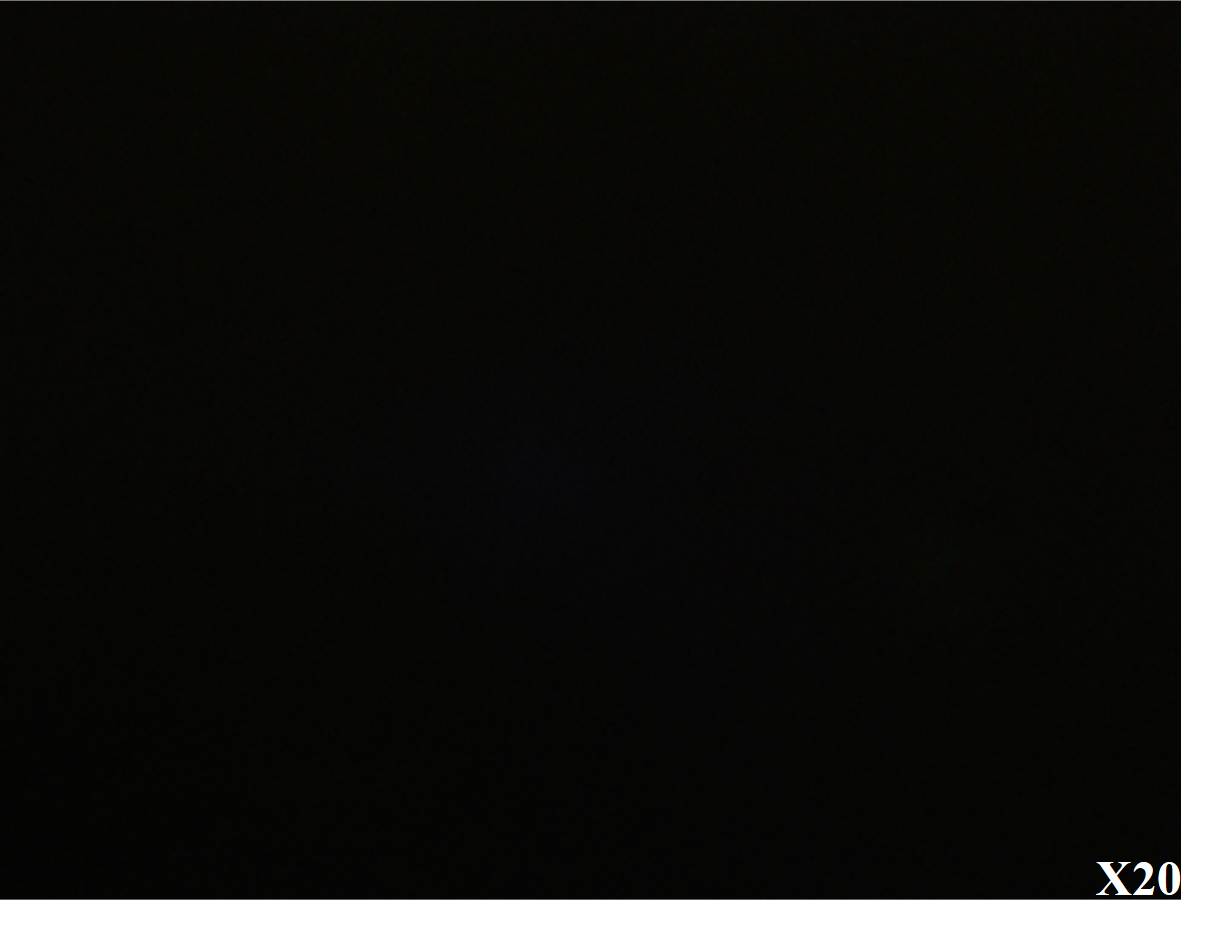


Fig. S1 POM observation of MT: Men=1:2


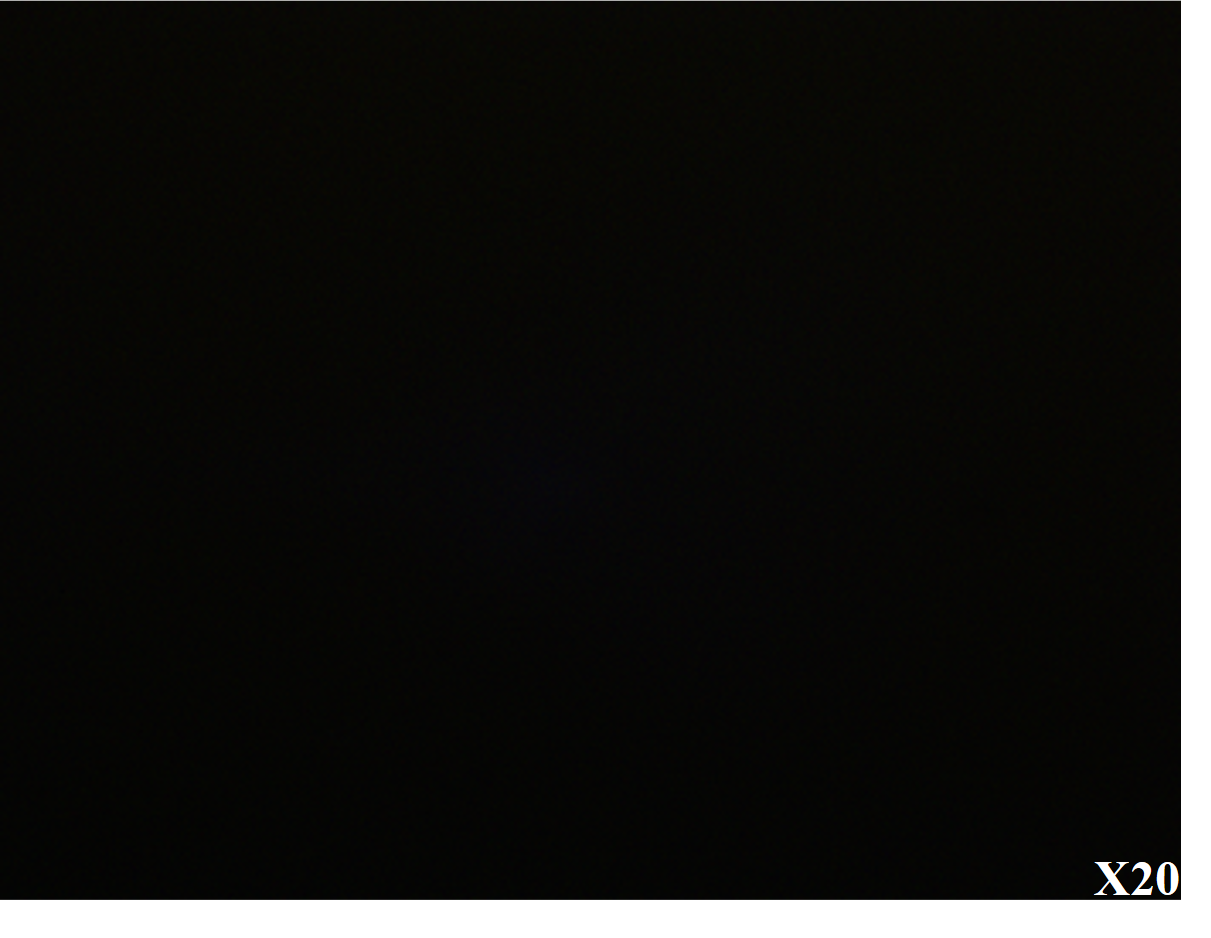


Fig. S2 POM observation of MT: Men=1:3


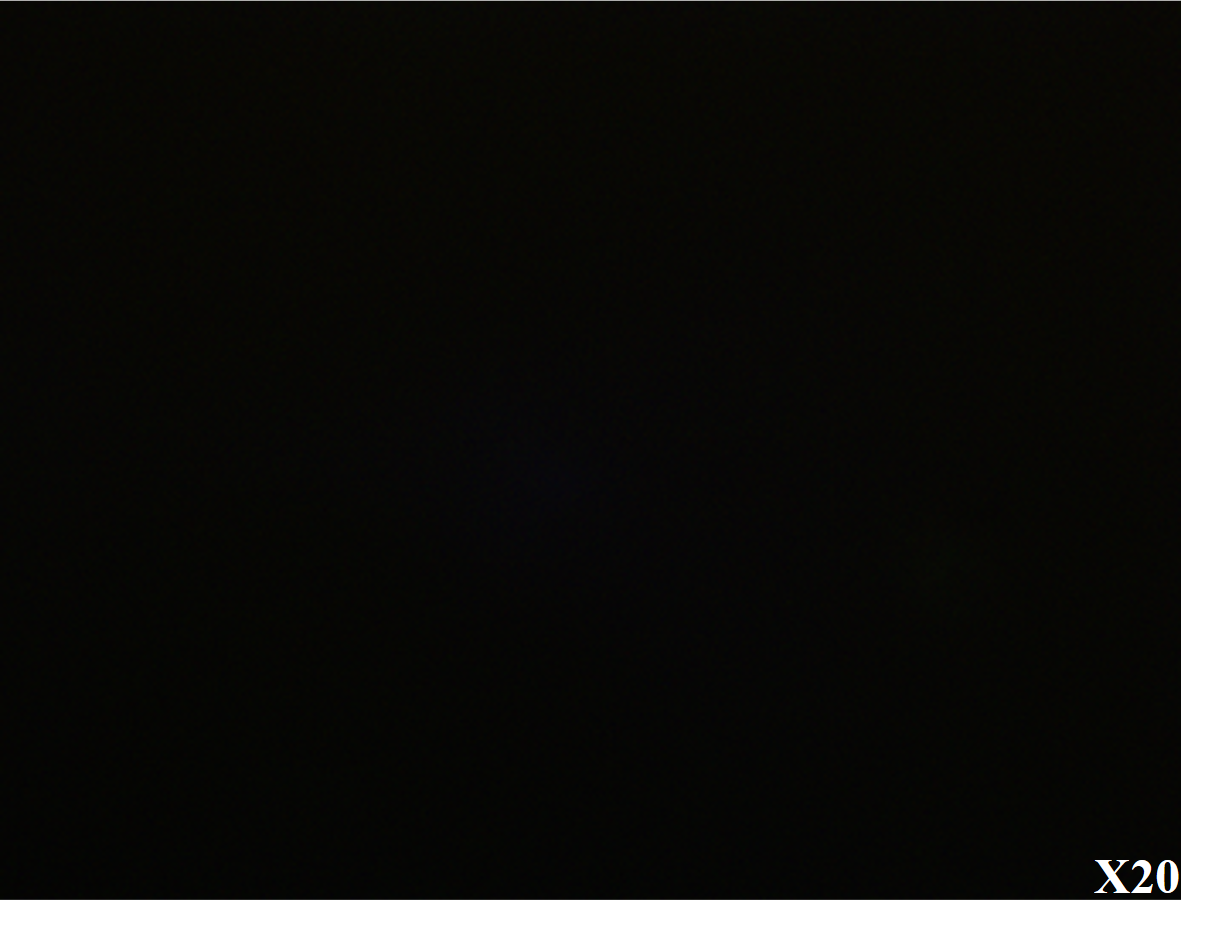


Fig. S3 POM observation of MT: Men=1:4


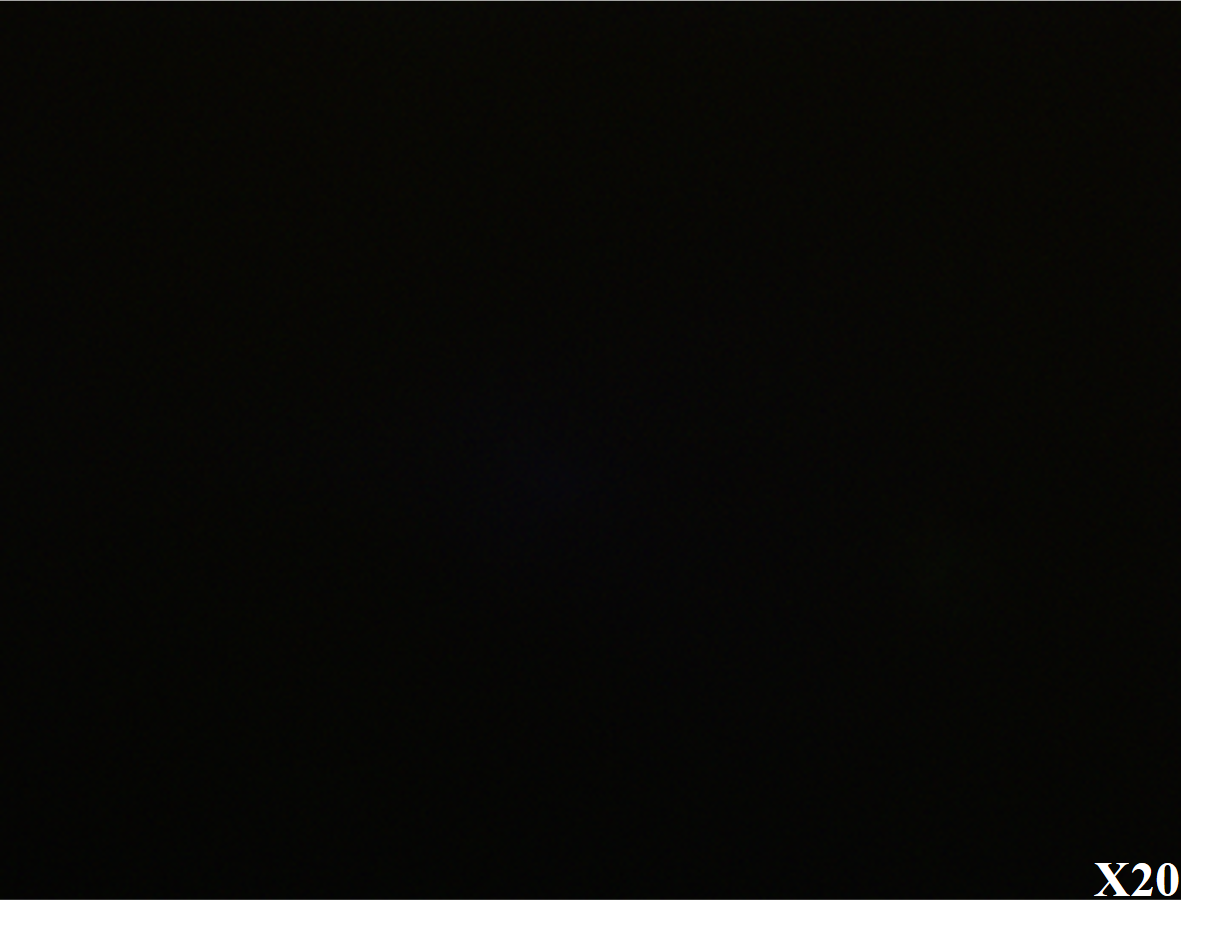


Fig. S4 POM observation of MT: Men=1:5


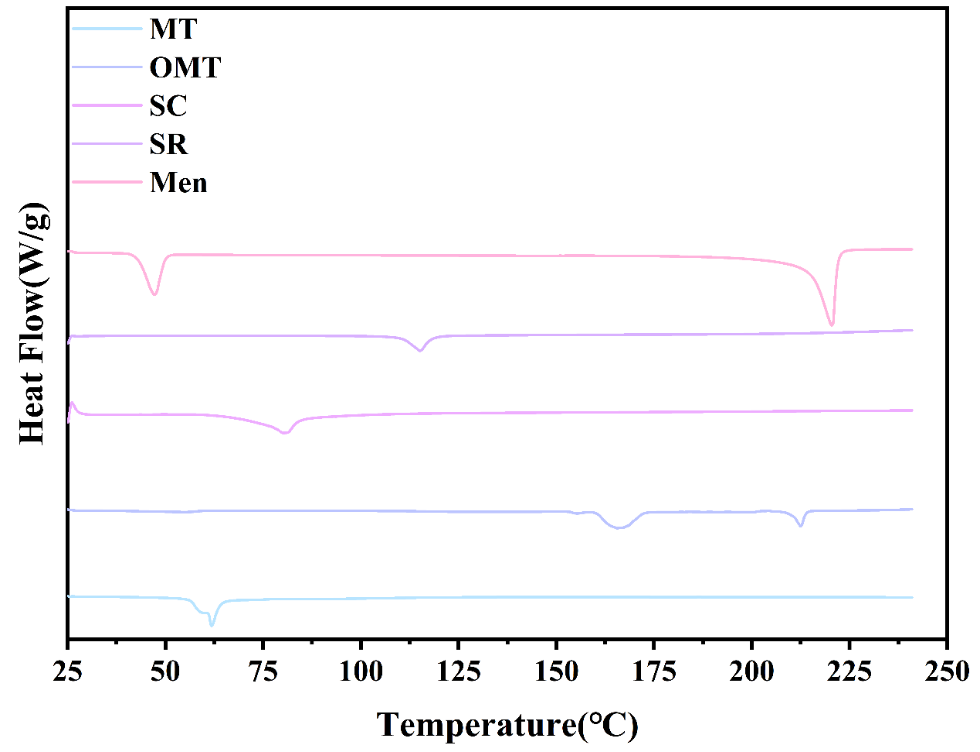


Fig. S5 DSC curve of pure component


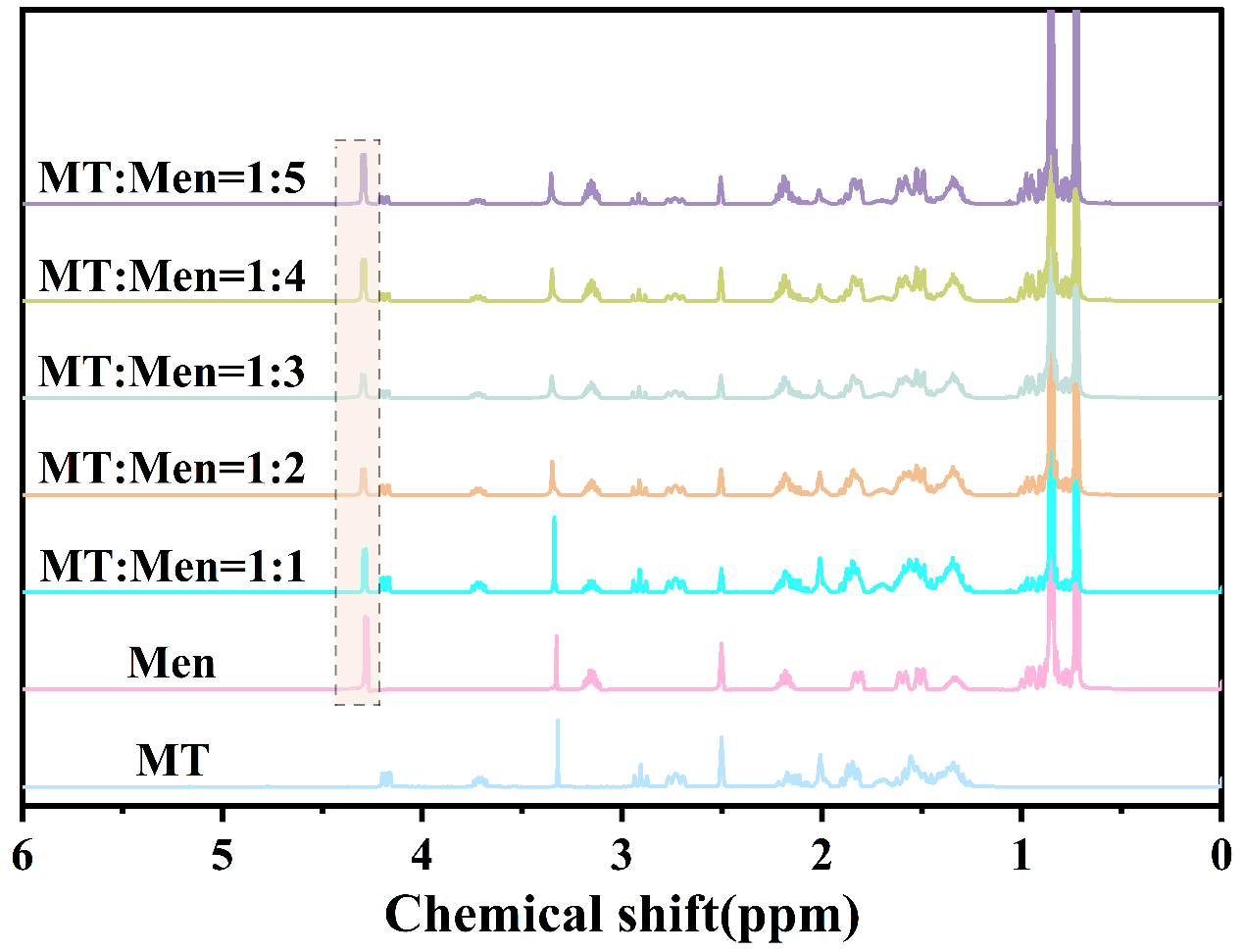


Fig. S6 ^1^H NMR spectra (400 MHz, DMSO-*d6*) of MT-Men DES and pure components


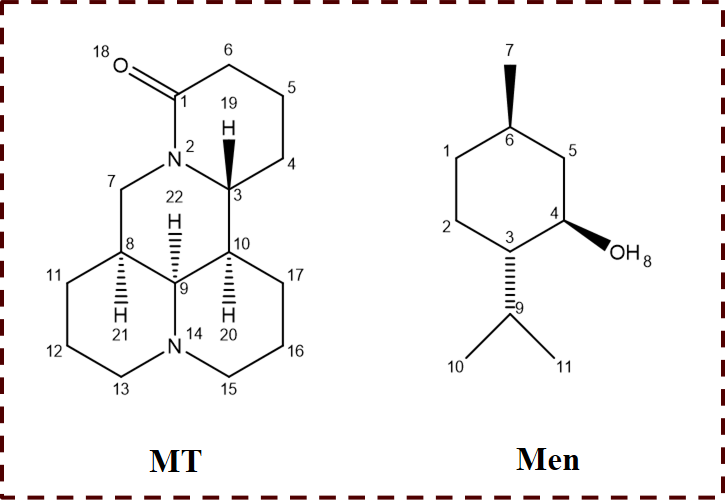


Fig. S7. The chemical structures and atomic numbers of MT and Men

Table S1. Summary of proton signal of MT and Men

| *δ*_H_ (ppm, DMSO-*d*_6_) | | | |
| --- | --- | --- | --- |
| MT | | Men | |
| H-4, H-11, H-17, H-12, H-16 | 1.853 | H-1, H-2 | 1.819 |
| H-5 | 2.002 | H-3 | 1.333 |
| H-6 | 2.730 | H-4 | 3.161 |
| H-7 | 4.179 | H-5 | 2.182 |
| H-13, H-15 | 2.907 | H-6 | 1.598 |
| H-19 | 3.711 | H-7, H-10, H-11 | 0.801 |
| H-20, H-21 | 1.447 | H-8 | 4.279 |
| H-22 | 2.145 | H-9 | 1.507 |

Table S2. Summary of proton signal of MT-Men (1:1) DES

| *δ*_H_ (ppm, DMSO-*d*_6_) | | | |
| --- | --- | --- | --- |
| MT | | Men | |
| H-4, H-11, H-17, H-12, H-16 | 1.848 | H-1, H-2 | 1.848 |
| H-5 | 2.004 | H-3 | 1.350 |
| H-6 | 2.732 | H-4 | 3.162 |
| H-7 | 4.186 | H-5 | 2.161 |
| H-13, H-15 | 2.912 | H-6 | 1.546 |
| H-19 | 3.717 | H-7, H-10, H-11 | 0.810 |
| H-20, H-21 | 1.546 | H-8 | 4.287 |
| H-22 | 2.161 | H-9 | 1.546 |

Table S3. Summary of proton signal of MT-Men (1:2) DES

| *δ*_H_ (ppm, DMSO-*d*_6_) | | | |
| --- | --- | --- | --- |
| MT | | Men | |
| H-4, H-11, H-17, H-12, H-16 | 1.840 | H-1, H-2 | 1.840 |
| H-5 | 2.006 | H-3 | 1.369 |
| H-6 | 2.734 | H-4 | 3.153 |
| H-7 | 4.187 | H-5 | 2.167 |
| H-13, H-15 | 2.913 | H-6 | 1.550 |
| H-19 | 3.718 | H-7, H-10, H-11 | 0.825 |
| H-20, H-21 | 1.550 | H-8 | 4.294 |
| H-22 | 2.167 | H-9 | 1.550 |

Table S4. Summary of proton signal of MT-Men (1:3) DES

| *δ*_H_ (ppm, DMSO-*d*_6_) | | | |
| --- | --- | --- | --- |
| MT | | Men | |
| H-4, H-11, H-17, H-12, H-16 | 1.839 | H-1, H-2 | 1.839 |
| H-5 | 2.006 | H-3 | 1.334 |
| H-6 | 2.734 | H-4 | 3.148 |
| H-7 | 4.188 | H-5 | 2.170 |
| H-13, H-15 | 2.915 | H-6 | 1.545 |
| H-19 | 3.720 | H-7, H-10, H-11 | 0.825 |
| H-20, H-21 | 1.545 | H-8 | 4.294 |
| H-22 | 2.170 | H-9 | 1.545 |

Table S5. Summary of proton signal of MT-Men (1:4) DES

| *δ*_H_ (ppm, DMSO-*d*_6_) | | | |
| --- | --- | --- | --- |
| MT | | Men | |
| H-4, H-11, H-17, H-12, H-16 | 1.831 | H-1, H-2 | 1.831 |
| H-5 | 2.013 | H-3 | 1.337 |
| H-6 | 2.734 | H-4 | 3.157 |
| H-7 | 4.190 | H-5 | 2.179 |
| H-13, H-15 | 2.915 | H-6 | 1.557 |
| H-19 | 3.720 | H-7, H-10, H-11 | 0.825 |
| H-20, H-21 | 1.557 | H-8 | 4.292 |
| H-22 | 2.179 | H-9 | 1.557 |

Table S6. Summary of proton signal of MT-Men (1:5) DES

| *δ*_H_ (ppm, DMSO-*d*_6_) | | | |
| --- | --- | --- | --- |
| MT | | Men | |
| H-4, H-11, H-17, H-12, H-16 | 1.833 | H-1, H-2 | 1.833 |
| H-5 | 2.008 | H-3 | 1.338 |
| H-6 | 2.735 | H-4 | 3.158 |
| H-7 | 4.190 | H-5 | 2.185 |
| H-13, H-15 | 2.917 | H-6 | 1.552 |
| H-19 | 3.722 | H-7, H-10, H-11 | 0.832 |
| H-20, H-21 | 1.552 | H-8 | 4.293 |
| H-22 | 2.185 | H-9 | 1.552 |


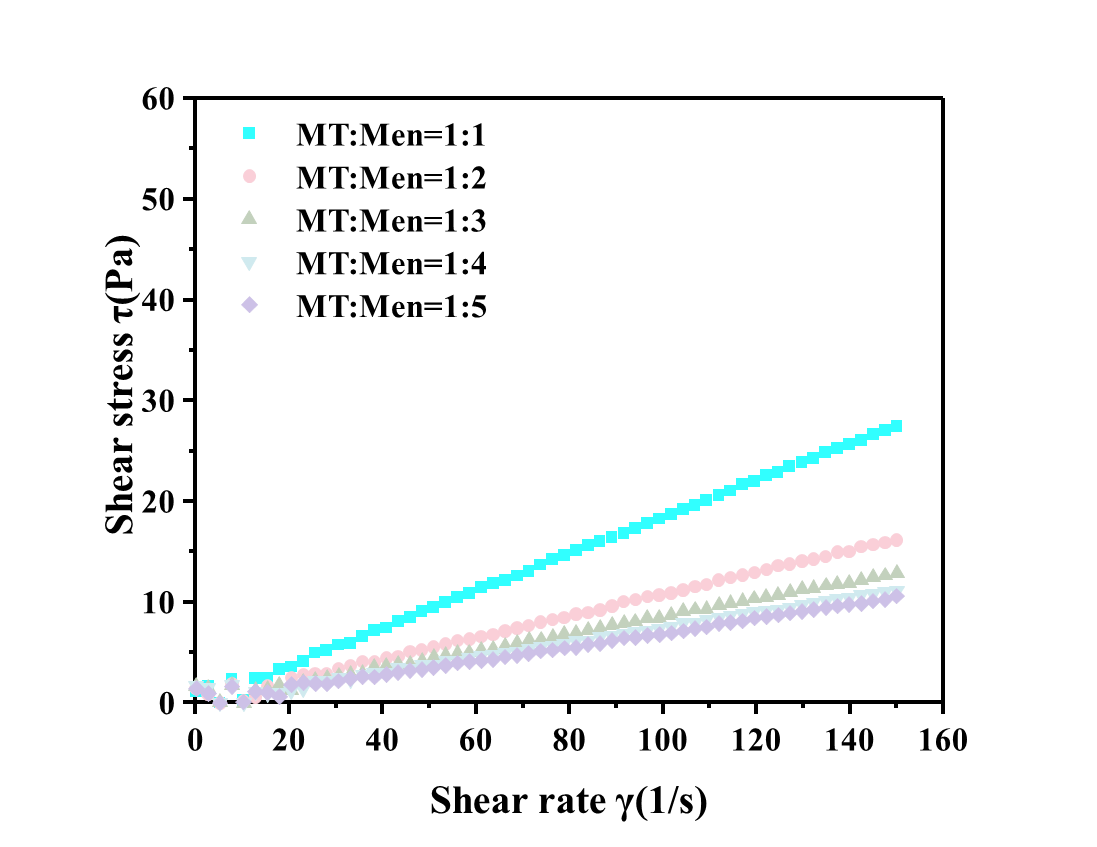


Fig. S8 Shear stress of MT-Men DES as a function of shear rate at 30 °C


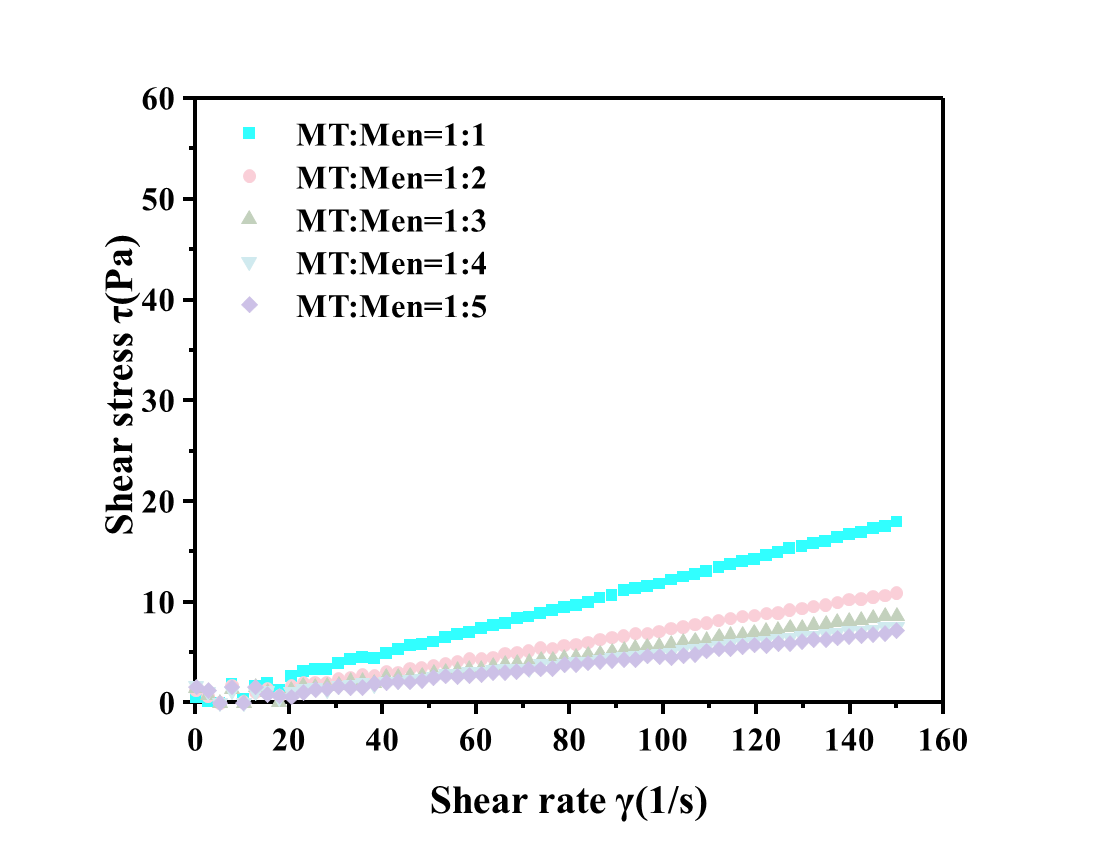


Fig. S9 Shear stress of MT-Men DES as a function of shear rate at 35 °C


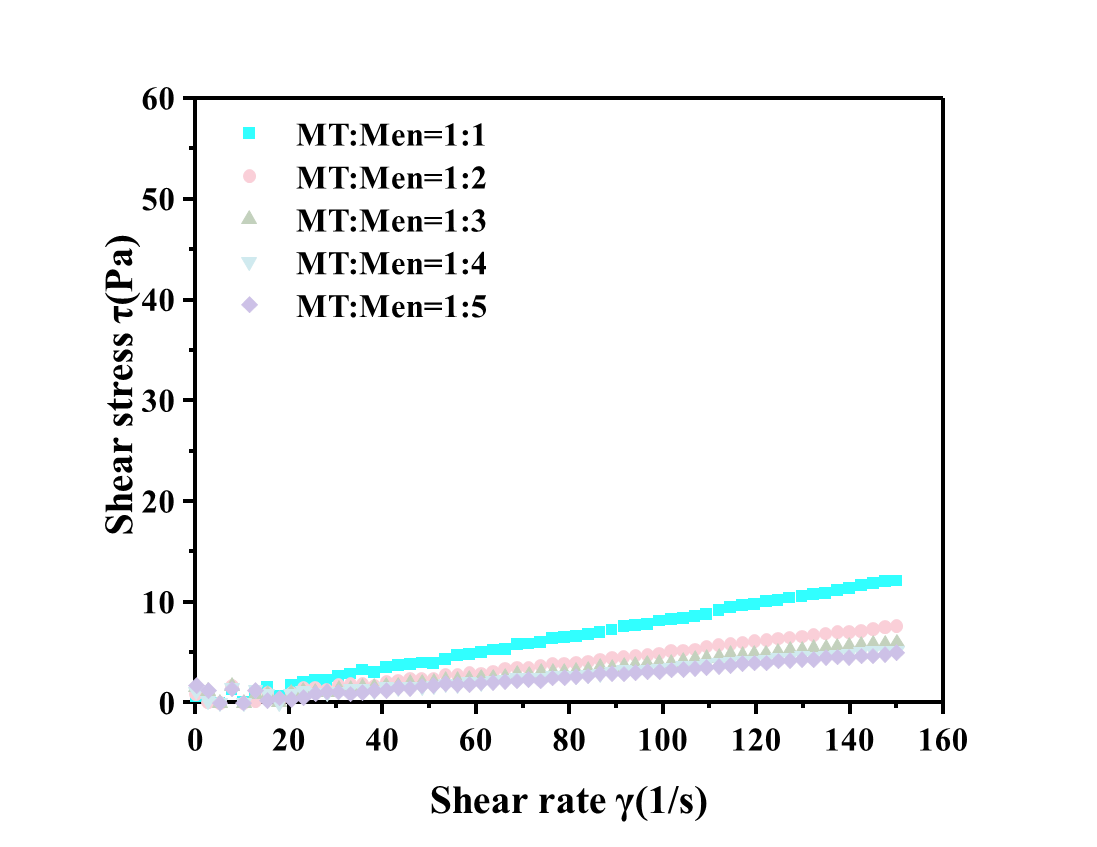


Fig. S10 Shear stress of MT-Men DES as a function of shear rate at 40 °C


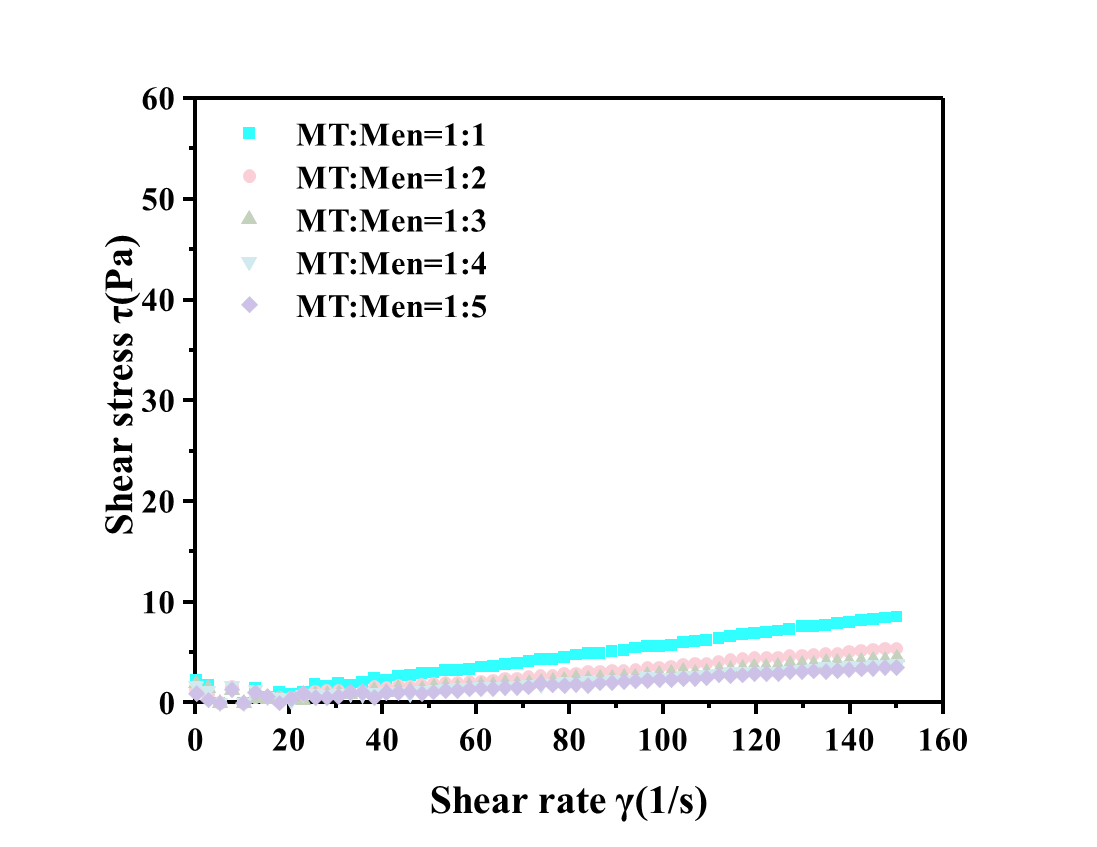


Fig. S11 Shear stress of MT-Men DES as a function of shear rate at 45 °C


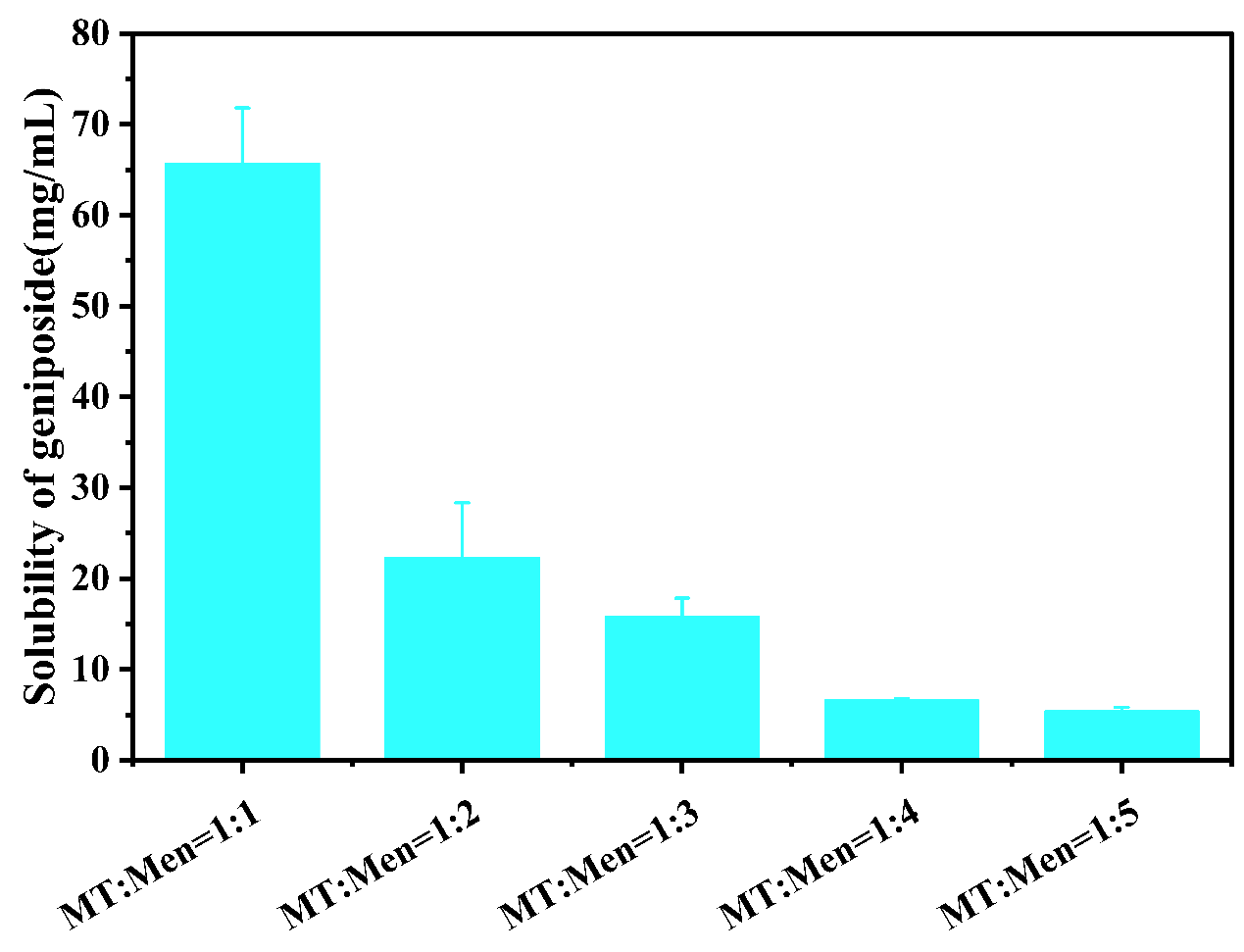


Fig. S12 Determination of the saturated solubility of GS in MT-Men DES with different molar ratios


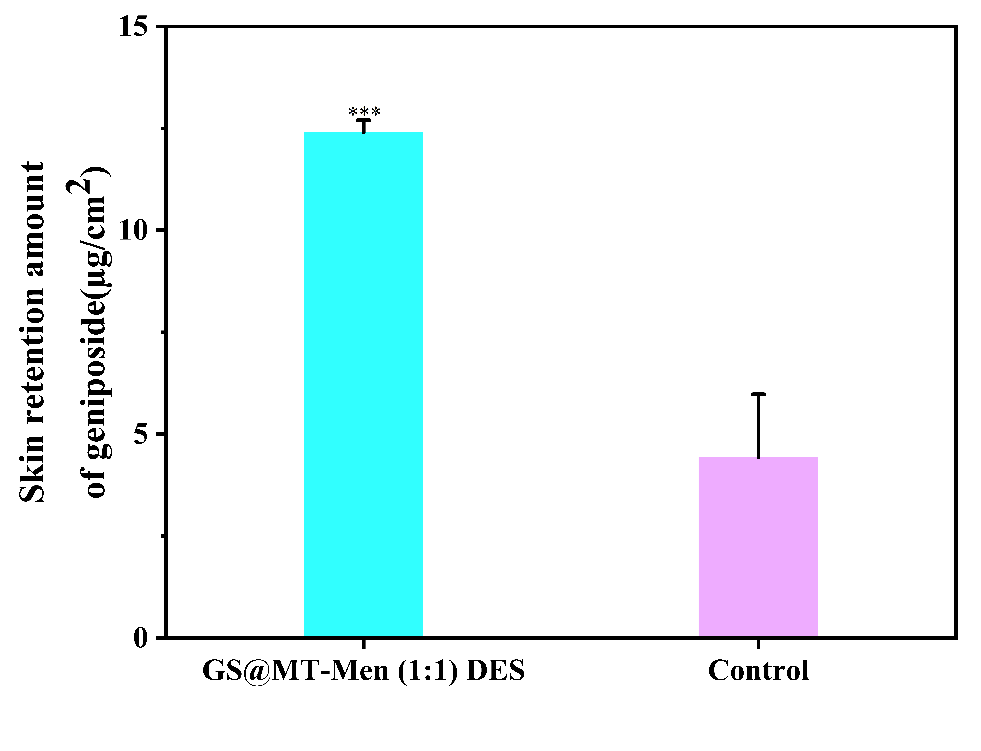


Fig. S13 Determination results of the 24-h ex vivo skin retention amount per unit area of GS in GS@MT-Men (1:1) DES


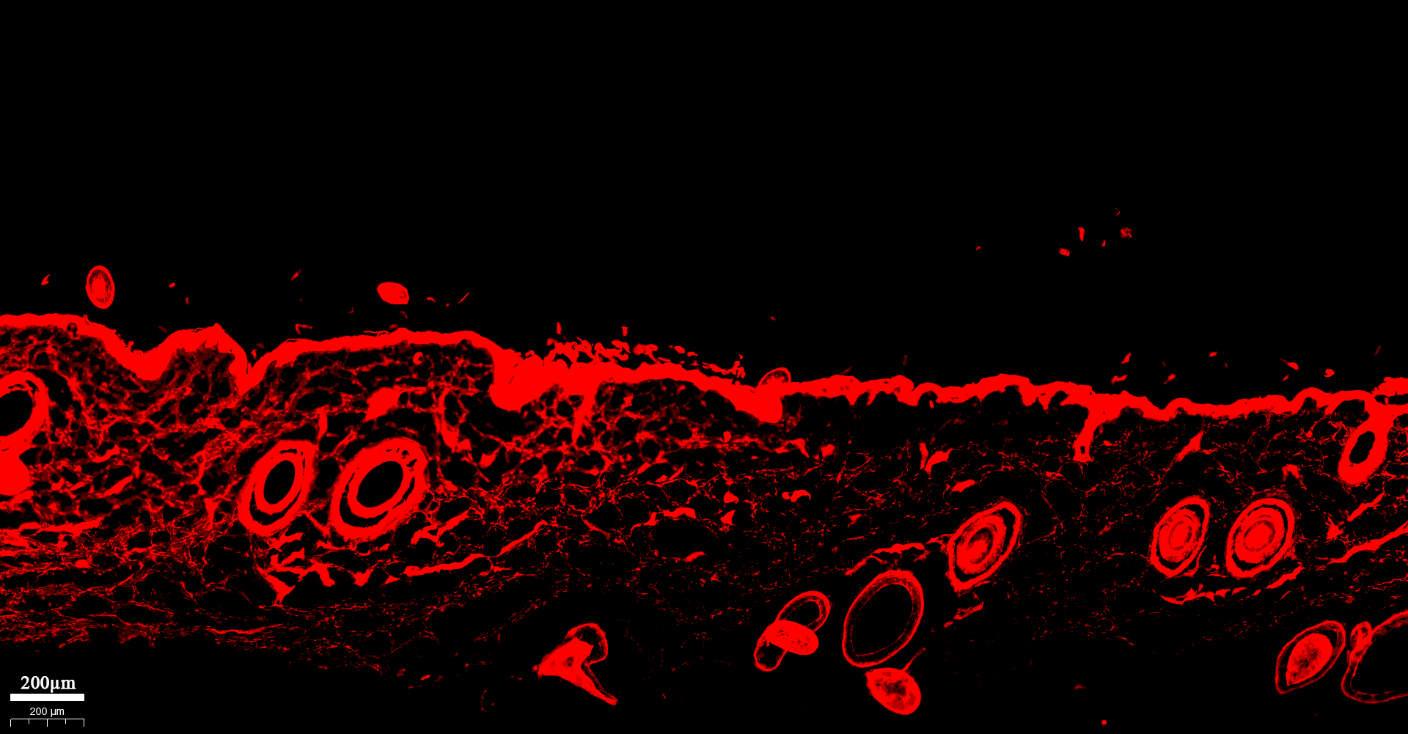


Fig. S14 Fluorescence microscopy of porcine skin treated with cy3-GS@MT-Men (1:1) DES


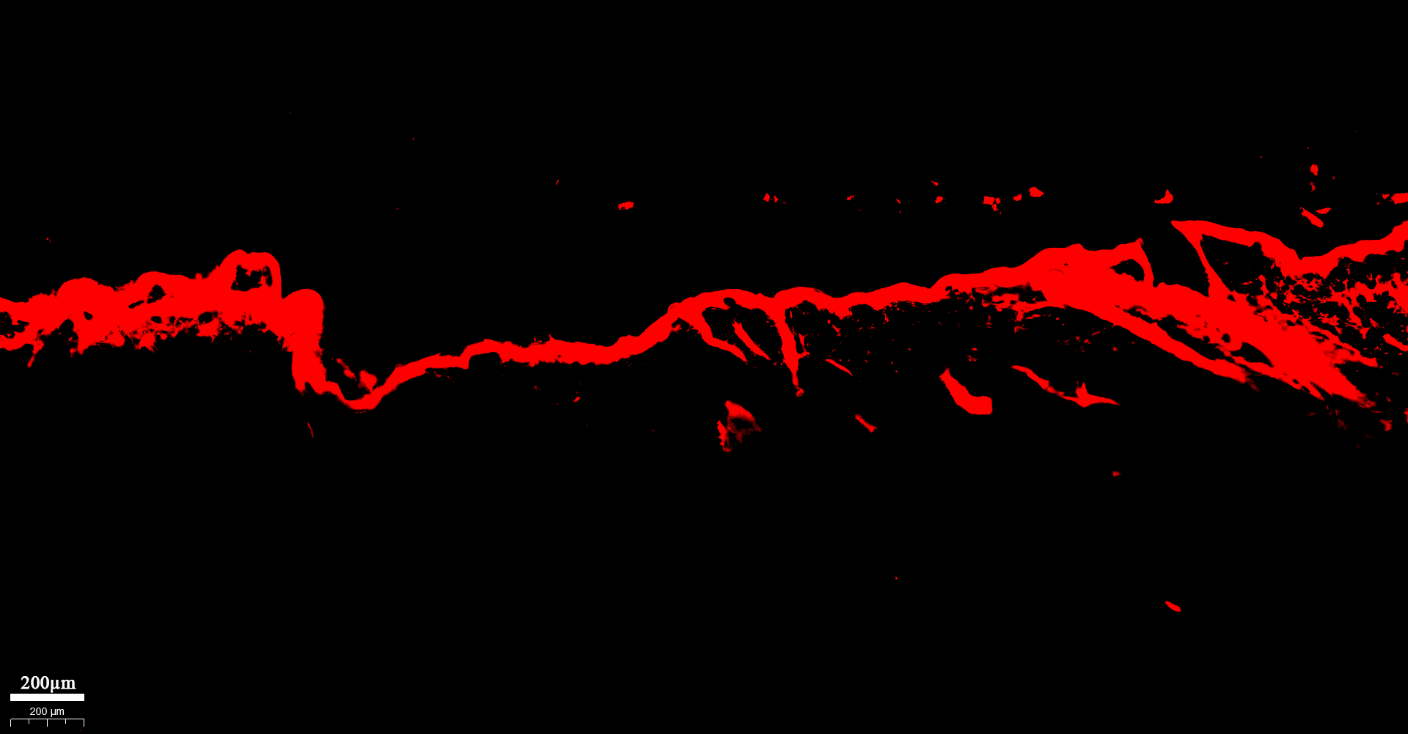


Fig. S15 Fluorescence microscopy of porcine skin treated with cy3-GS aqueous solution


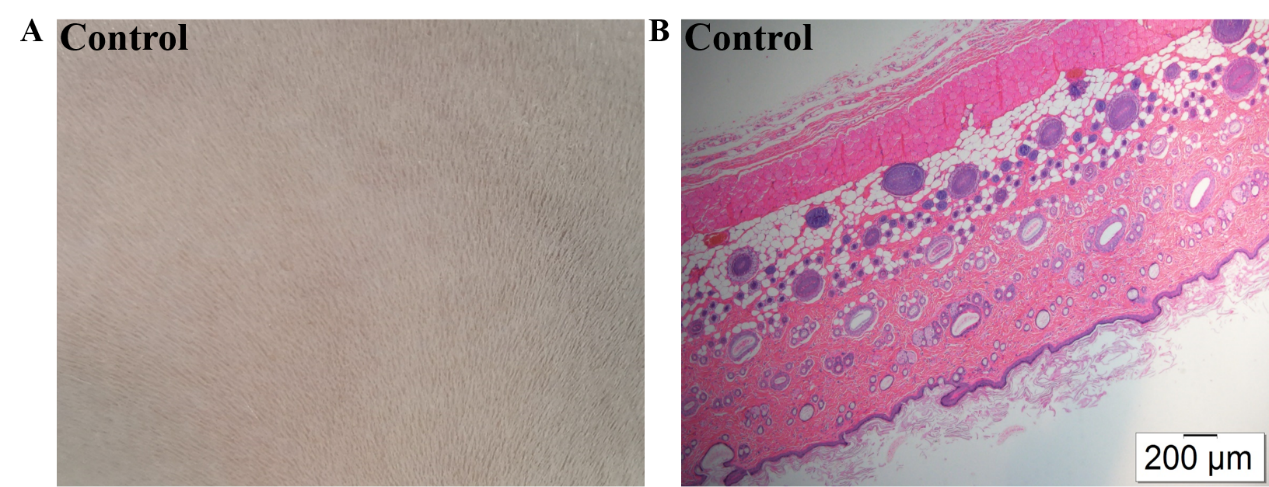


Fig. S16 Histopathological examination of normal rat skin. Visual observation (A) and H&E staining results of skin sections (B) of normal skin in rats (n=6)
